# Hierarchically Rough rGO/HAp Nanocoatings on SLM Ti–6Al–4 V Implants for Enhanced Biointegration

**DOI:** 10.1002/smll.75211

**Published:** 2026-08-21

**Authors:** Muhammad Usama Zaheer, Vahid Jahed, Rajveer Singh Rajaura, Fatemeh Rastegar Adib, Muhammad Hassan Razzaq, Gökhan Demirel, Mobarak Abu Mraheil, Thaqif El Khassawna, Mehmet Fatih Aycan, Yogendra Kumar Mishra

**Affiliations:** ^1^ Smart Materials NanoSYD Mads Clausen Institute University of Southern Denmark Sønderborg Denmark; ^2^ Department of Mechanical Engineering Institute for Graduate School of Natural and Applied Sciences Gazi University Ankara Türkiye; ^3^ Additive Manufacturing Technologies Application and Research Center – EKTAM Gazi University Ankara Türkiye; ^4^ Experimental Trauma Surgery Faculty of Medicine Justus‐Liebig‐University Giessen Germany; ^5^ School of Engineering and Materials Science Queen Mary University of London London UK; ^6^ Institute for Medical Microbiology Justus‐Liebig‐University Giessen Germany; ^7^ Department of Chemistry Faculty of Science Gazi University Ankara Türkiye

**Keywords:** biointegration, cytocompatibility, hierarchical morphology, orthopedic SLM implants, rGO/HAp nanocoating

## Abstract

Additive manufacturing of Ti–6Al–4V implants enables patient‐specific design, but process‐induced surface and microstructural variations can strongly affect biological performance. In this work, Ti64 samples with varying volumetric energy densities (VEDs) were fabricated by selective laser melting to examine the combined effects of processing and surface modification on cell response and antibacterial behavior. The specimens were surface activated by controlled acid etching and coated with hydroxyapatite/reduced graphene oxide (HAp/rGO) composite via electrophoretic deposition. Surface morphology, chemistry, and porosity were assessed using Scanning Electron Microscopy, X‐ray Photoelectron Spectroscopy, profilometry, micro‐CT. Micro‐CT showed higher internal porosity at lower VED, while a balance between densification and structural integrity was achieved at VED of 107 J mm^−^^3^. In vitro cytocompatibility demonstrated >90% viability for all samples, meeting ISO 10993‐5 requirements. Despite this, cell attachment depends on VED, the E63 condition exhibited the most uniform coverage after HAp/rGO coating, indicating an improved surface–cell interactions at lower VED. Antibacterial assays showed VED dependence; high‐VED‐E320 samples produced the highest biofilm formation even after coating, whereas low and intermediate VED conditions significantly reduced bacterial adhesion. Overall, biological functionality is observed to be governed by both coating and SLM parameters, with 107 J·mm^−^^3^ offering the best combined structural, cytocompatible, and antibacterial performance.

## Graphical abstract

This study investigates HAp/rGO bioactive coatings on Ti‐6Al‐4V fixation implants fabricated by selective laser melting with systematically varied volumetric energy density to tailor the as‐built surface state. Composite coatings were deposited by electrophoretic deposition and characterized using micro‐CT, profilometry, FESEM/EDS, and detailed XPS to elucidate interfacial chemistry. A half‐coated/half‐uncoated specimen design enabled direct comparison under identical culture conditions. Biological assays evaluated cell adhesion, viability, proliferation, and antibacterial performance.

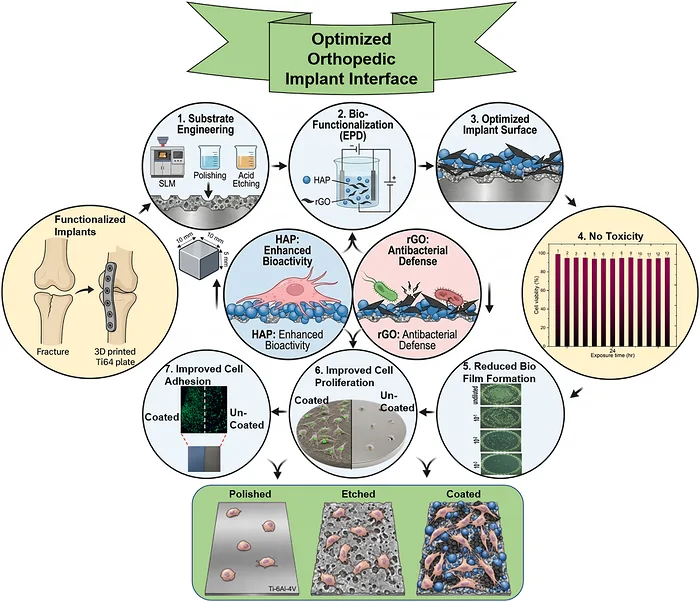

**Abbreviations**
AM: Additive Manufacturing
MSC: Bone Marrow–Derived Mesenchymal Stem Cells
Ca/P: Calcium‐to‐Phosphorus Ratio
EDS: Energy‐Dispersive X‐ray Spectroscopy
EPD: Electrophoretic Deposition
FESEM: Field Emission Scanning Electron Microscopy
FTIR: Fourier Transform Infrared Spectroscopy
HAp: Hydroxyapatite
HNO_3_
: Nitric Acid
HF: Hydrofluoric Acid
micro‐CT: Micro–Computed Tomography
PEO: Plasma Electrolytic Oxidation
rGO: Reduced Graphene Oxide
SBF: Simulated Body Fluid
SLM: Selective Laser Melting
Ti64: Ti‐6Al‐4 V
TiO_2_
: Titanium Dioxide
VED: Volumetric Energy Density
XPS: X‐ray Photoelectron Spectroscopy

## 1 Introduction

The clinical success of orthopedic and dental implants hinges on osseointegration, typically quantified by metrics such as bone–implant contact (BIC), interfacial shear strength, and implant fixation stability, which collectively reflect the structural and functional coupling between living bone and the implant surface [1]. Following *Brånemark's* foundational work in the 1970s, osseointegration was initially framed as a predominantly material‐driven phenomenon, with implant success attributed mainly to bulk biocompatibility and the absence of adverse host reactions. However, contemporary evidence frames osseointegration as an active, immune‐regulated foreign body response, where early macrophage‐driven signaling and subsequent osteoprogenitor recruitment shape a mineralized interface that stabilizes the implant within host tissue [2, 3]. Among design variables governing integration, surface topography and surface chemistry act as coupled regulators of early protein adsorption and cell–material signaling, thereby influencing adhesion, osteogenic differentiation, and long‐term osseointegration [4, 5, 6]. The interplay between microroughness (commonly in the ∼1–2 µm Ra regime) and nanoscale features modulates adsorption and conformation of adhesion proteins (e.g., fibronectin/vitronectin), driving integrin engagement, osteogenic differentiation, and ultimately bone–implant bonding [4, 7].

Hydroxyapatite (HAp) and graphene‐derived coatings have each been explored to enhance titanium biointegration; however, HAp coatings are often limited by brittleness and interfacial delamination, whereas graphene‐based layers alone lack intrinsic osteoconductive cues, motivating hybrid HAp–graphene architectures [8, 9, 10, 11]. HAp is osteoconductive, but coatings can be mechanically fragile and prone to microcracking or interfacial debonding under shear, cyclic fatigue, and fretting‐type loading typical of load‐bearing implant environments [12], which has driven interest in reinforcing HAp with graphene oxide (GO) or reduced graphene oxide (rGO) [13]. For example, Bulbul and Aksakal et al. demonstrated that introducing rGO into an HAp matrix (sol–gel) on Ti‐64 produced a more homogeneous morphology with reduced microcrack density and improved apparent coating continuity and adhesion compared with bare HAp coatings. Their work was performed on laser‐treated, conventionally prepared Ti64 surfaces and relied primarily on Raman, XRD, and FESEM for characterization rather than deeper chemical‐state analysis of the coating/substrate interface [14]. Related studies on HA/rGO produced by plasma electrolytic oxidation (PEO), for instance, explicitly characterize phase, morphology, functional groups and composition via XRD, SEM, ATR‐FTIR, EDS, and Raman (and optimize voltage/current density), but are unable to provide the type of detailed XPS chemical‐state mapping needed to mechanistically understand the interfacial chemistry and bonding environments in HA/rGO–Ti64 systems [15]. Accordingly, a key unresolved gap is the chemical‐state structure at the HAp/rGO–Ti64 interface (e.g., Ca–P environments, Ti–O contributions, graphene‐related C─O/C═O states) and the extent to which these interfacial states correlate with biological performance [16, 17, 18].

In parallel, electrophoretic deposition (EPD)‐based composite coatings have shown strong biological promise on titanium substrates. Suo et al. [19]. fabricated a graphene oxide/chitosan/hydroxyapatite (GO/CS/HA) coating by EPD on Ti substrates and reported improved wettability, bonding strength, and enhanced in vitro cell–material interactions, supported by in vivo osseointegration evidence. Likewise, Baheti et al., [20] deposited GO, HAp, and HAp–GO composite coatings (2 and 5 wt% GO) via electrophoresis deposition on Ti implant surfaces and observed improved roughness/hydrophilicity alongside enhanced BMSC adhesion, spreading, proliferation, and osteogenic differentiation compared with uncoated Ti or single‐component coatings [21]. While these studies establish the biomedical potentials of HAp–graphene composite systems deposited by EPD, they are primarily demonstrated on Ti substrates prepared through conventional routes rather than on Ti64 components manufactured by selective laser melting (SLM) [22, 23]. Moreover, these studies do not systematically resolve how AM‐specific variables such as volumetric energy density (VED) precondition surface morphology/oxide state and thereby influence subsequent HAp/rGO deposition, interface chemistry, and biological response [20, 24]. Another and increasingly important gap is that most HAp/GO(rGO) coating studies have been developed without explicitly integrating the substrate “manufacturing history” into the coating design. Additive manufacturing particularly SLM can generate markedly different near‐surface microstructures, oxide characteristics, and nanoscale roughness features compared with conventionally machined Ti64, and these factors can significantly influence coating adhesion, coating continuity, and ultimately cell biological response. To the best of our knowledge, prior HAp/GO(rGO) work rarely treats SLM process parameters as deliberate levers for downstream coating performance.

In the present work, we address these limitations by integrating additive manufacturing, surface‐morphology control, and composite coating design into a single experimental framework. The Ti64 fixation‐implant specimens were fabricated by SLM while systematically varying VED to tune the pristine surface condition prior to coating, and then a bioactive HAp/rGO composite coating was deposited by EPD, leveraging EPD's suitability for conformal coating of complex implant geometries demonstrated in prior EPD‐based implant coating studies. To strengthen the biological interpretability beyond the conventional “separate coated *vs* separate uncoated sample” approach, each specimen was designed to be half‐coated and half‐uncoated, enabling within‐sample, side‐by‐side comparison of coated versus uncoated regions under identical culture conditions, thereby reducing inter‐sample variability in biological readouts. Finally, the coating/substrate system was investigated using micro‐CT, profilometry, FESEM/EDS, and, critically, a detailed XPS chemical‐state analysis. In vitro assays were performed to assess early‐stage cell–surface interactions, including cell adhesion, viability, and proliferation on HAp/rGO‐coated regions compared with uncoated regions. In addition, antibacterial testing was conducted to assess the coating's ability to inhibit bacterial attachment and growth, providing an integrated evaluation of its biological response and potential to reduce implant‐associated infection risks while supporting favorable cellular activity. (Supplementary Information Figures S1 and S2, shows actual SLM samples prepared and complete manufacturing and coating schematic).

## 2 Experimental Section

### 2.1 Additive Manufacturing of Ti–6Al–4 V (Ti64)

Gas‐atomized Ti64 alloy powder with a spherical morphology and a particle size distribution between 20 µm and 40 µm was procured from General Electric (GE, USA). Small cubic Ti64 specimens (10 × 10 × 5 mm^3^) were directly fabricated on the build plate via SLM using an Ermaksan ENAVISION 250 system located at the Additive Manufacturing Technology Application and Research Center (EKTAM), Gazi University, Ankara, Turkey. The VED was controlled by varying the laser scan speed while keeping the laser power, hatch distance, and layer thickness constant. The process parameters used for SLM of Ti64 are summarized in Table 1 and VED calculated by Equation 1. Four samples, denoted as E63, E107, E200, and E320, correspond to VED values of 63, 107, 200, and 320 J mm^−^^3^, respectively. The selected VED levels were down‐selected from a broader preliminary SLM processing window based on visual and SEM surface observations. These four conditions were chosen because they represented the distinct surface morphologies observed within the investigated range. The low VED condition produced insufficient melting features, including partially melted particles and lack‐of‐fusion‐prone regions, whereas the high VED condition resulted in excessive melting, remelted regions, balling/spatter‐like particles, and surface instability. Intermediate VED values represented stable melt‐pool formation and slightly over‐melted behavior. VED values below 63 J mm^−^^3^ were not considered suitable because they resulted in poor consolidation and discontinuous melt tracks, while VED values above 320 J mm^−^^3^ promoted excessive heat accumulation, severe over‐melting, spattering, and dimensional instability. Therefore, the selected VED range of 63–320 J mm^−^^3^ was used to capture the major SLM‐induced surface conditions relevant to subsequent acid etching and coating deposition.

(1) $$VED=\frac{P}{\nu \;\times \;h\;\times t}$$

where *P* is the laser power (W), *v* is the scan speed (mm s^−^^1^), *h* is the hatch spacing (mm), and *t* is the layer thickness (mm). The VED represents the energy input per unit volume of material and serves as a key indicator of melting behavior during the SLM process. Each sample was subjected to sequential grinding with SiC abrasive papers up to 2000 grit, followed by mirror‐finish polishing using diamond suspensions down to 1 µm. To introduce controlled surface roughness, the polished specimens were chemically etched in an aqueous solution of nitric acid (HNO_3_) and hydrofluoric acid (HF) for different durations (5, 10, 30, 60, 360, 720, 1080, and 1440 min). After etching, samples were rinsed with deionized water and dried under nitrogen flow.

**TABLE 1:** Process parameters and corresponding volumetric energy densities (VED) for SLM‐fabricated Ti–6Al–4 V samples. The scan speed was varied to adjust the VED, while laser power, hatch distance, and layer thickness were kept constant.

Sample	Power (W)	Scan speed (mm s^−^^1^)	Hatch distance (mm)	Layer thickness (mm)	VED (J mm^−^^3^)	Explanation
E320	370	241	0.12	0.04	320	Excessive energy
E200	370	385	0.12	0.04	200	Slightly over‐melted
E107	370	729	0.12	0.04	107	Stable melt pool
E63	370	1215	0.12	0.04	63	Insufficient energy

### 2.2 EPD‐Based Deposition of HAp/rGO Composite Coatings

Reduced graphene oxide (rGO) powder with a purity of 99% and a thickness of 2–5 layers (particle size: 1–10 µm) was procured from Nanografi Nanotechnology Inc. (Ankara, Turkey). Hydroxyapatite (HAp) nanopowder with a purity of ≥ 95% and an average particle size of approximately 50 nm was also obtained from the same supplier. A HAp/rGO electrophoretic bath was prepared in ethanol (low‐conductivity, particle‐stabilizing medium) containing HAp at 1.5 g L^−^^1^ and rGO at 0.3 g L^−^^1^; polyethyleneimine (PEI, 0.1 g L^−^^1^) was added as a dispersant/charge modifier. The suspension pH* was adjusted to ∼5.5 with acetic acid and water, then sonicated (ultrasonic bath, 40 kHz, 30 min) to break agglomerates and yield a dispersion stable for ≥30 min. EPD was carried out at room temperature with the Ti64 specimen used as the cathode and a stainless‐steel counter electrode used as the anode, positioned 1.5 cm away from the Ti64 substrate. This polarity was selected because the HAp/rGO suspension exhibited a net positive particle charge, causing the positively charged particles to migrate toward and deposit on the negatively charged Ti64 cathode. A potential of 30 V was applied for 3 min to obtain a continuous composite coating. All samples were half masked with Kapton tape before EPD in order to produce half coated samples. The samples were air‐dried for 24 h and sintered at 500 °C for 1 h under Ar gas, followed by controlled cooling at ∼1 °C min^−^^1^ to minimize thermal‐stress cracking and improve coating adhesion/crystallinity, while limiting rGO oxidation in a vacuum tube furnace [25].

### 2.3 Microstructural, Morphological, and Chemical Analysis

Cubic coupons (10 × 10 × 5 mm^3^) were produced specifically to evaluate internal features without sectioning the parts. The as‐built cubes were scanned using a high‐resolution micro‐computed tomography system (Zeiss Xradia 510, Carl Zeiss, Germany). Micro‐CT enabled non‐destructive three‐dimensional visualization and quantification of the internal architecture, including pore volume fraction and spatial pore distribution. These measurements were used to compare how variations in VED during SLM influence the formation and localization of internal voids in the fabricated samples, and tests performed at EKTAM, Gazi University, Ankara, Turkey. Surface/topography metrics were obtained by stylus profilometry, and chemical characterization of the coating was performed using FTIR and XPS. Surface roughness was measured using a Dektak 150 profilometer (Veeco Instruments, USA) equipped with a diamond‐tipped stylus, providing a vertical resolution of 6 Å and a scan length up to 55 mm, while FTIR spectra were recorded using a VERTEX 80 spectrometer (Bruker Optics, Germany) equipped with a DLATGS detector, KBr beam splitter, and diamond ATR accessory at Gazi University, Türkiye. The system operated in the 15,500–350 cm^−^^1^ range with a spectral resolution of 4 cm^−^^1^ to identify surface functional groups and chemical bonds on the samples. Raman spectroscopy was performed to identify the characteristic vibrational bands of HAp and rGO in the composite coating at Gazi University Türkiye. Raman spectra were collected over the wavenumber range of approximately 400–3200 cm^−^^1^ using a 532 nm excitation laser. Prior to peak analysis, the spectra were baseline‐corrected to remove background fluorescence and normalized for comparison. The HAp‐related phosphate vibration was identified around 960 cm^−^^1^ and assigned to the ν_1_ PO_4_^3^^−^ mode. The characteristic D and G bands of rGO were identified at approximately 1348–1351 cm^−^^1^ and 1586–1588 cm^−^^1^, respectively. The D band is associated with defect‐induced vibrations and disordered carbon, whereas the G band corresponds to the in‐plane vibration of sp^2^‐bonded carbon atoms. The XPS measurements were performed using a Thermo Scientific K‐Alpha spectrometer at the School of Engineering and Materials Science, Queen Mary University of London, United Kingdom. The samples were mounted on the XPS sample holder using double‐sided conductive carbon tape to minimize charging effects. The XPS spectra were acquired using a monochromated Al Kα X‐ray source with a photon energy of 1486.6 eV, operated at 36 W, and a spot size of 400 µm. Microstructural and morphological analyses were performed using a Hitachi SU8700 ultrahigh‐resolution Schottky field‐emission scanning electron microscope (Hitachi High‐Tech, Japan). The SU8700 supports imaging over an accelerating voltage range of 0.1–30 kV and can resolve features as small as 0.6 nm at 15 kV (0.8 nm at 1 kV). It is equipped with multiple detectors, high probe current capability (up to 200 nA), and a 5‐axis motorized stage accommodating specimens up to 150 mm in diameter. The instrument also allows low‐voltage imaging without stage bias, which is beneficial for beam‐sensitive samples. At Gazi University, the SU8700 was used to acquire high‐magnification secondary electron (SE) and backscattered electron (BSE) images of the as‐fabricated and processed specimens, enabling detailed examination of surface morphology and interface features. Figure 1a–c provides an overview of the processing strategy and the functional rationale of the surface modification approach adopted in this work. As illustrated schematically, the resulting surface is expected to exhibit a dual‐action behavior, in which HAp promotes cell adhesion and viability, while rGO imparts antibacterial activity by suppressing the bacterial attachment and proliferation. This combined architecture is designed to simultaneously enhance osseointegration and reduce infection risk at the implant interface.

**FIGURE 1:**
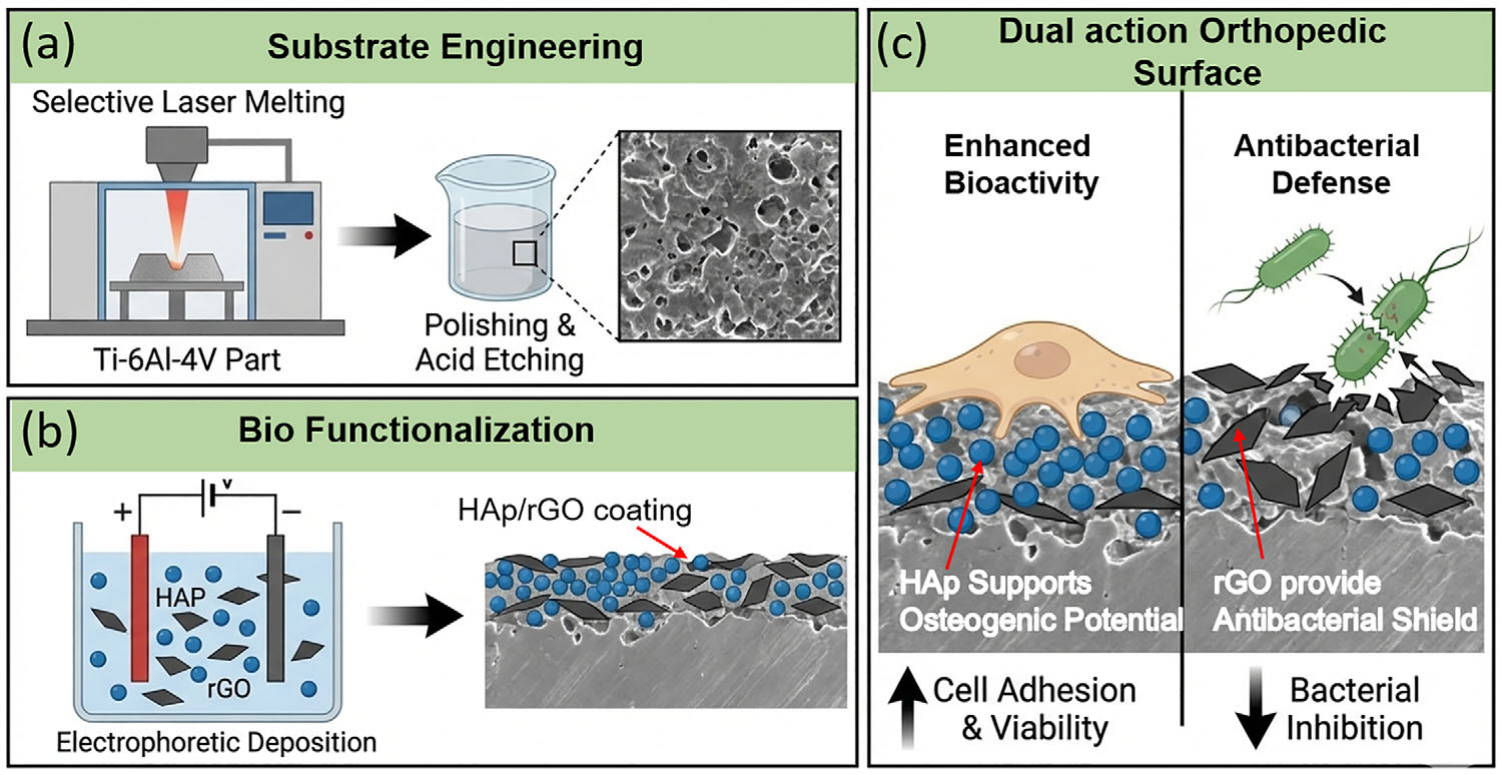
(a,b) Schematic of the fabrication workflow for HAp/rGO‐coated Ti64, including SLM processing, polishing, and acid etching, followed by EPD of HAp and rGO to form a hybrid surface layer. (c) Illustration of the dual‐action functionality of the HAp/rGO coating, where HAp enhances bioactivity by promoting cell adhesion and viability, while rGO provides antibacterial protection through inhibition of bacterial attachment and proliferation, resulting in a multifunctional orthopedic surface.

### 2.4 Coating Adhesion Evaluation

Progressive‐load scratch testing was performed to evaluate the adhesion integrity of the HAp/rGO coating on the Ti64 substrate. The test was conducted using a micro‐scratch tester equipped with a Rockwell C diamond indenter with a tip radius of 200 µm. The normal load was linearly increased from 1 to 35 N over a scratch length of 5 mm at a scratch speed of 5 mm/min. At least three scratches were performed for each sample. The critical loads were determined by correlating friction‐force signals. The first cohesive cracking, initial coating delamination, and complete coating removal were defined as L_c1_, L_c2_, and L_c3_, respectively.

### 2.5 Cell Isolation

hBMSCs were isolated from femoral head bone samples obtained from patients undergoing orthopedic surgery after written informed consent was provided. Bone fragments containing bone marrow were collected under sterile conditions and washed thoroughly with PBS to remove blood and debris. The samples were then minced into small pieces and digested with a 0.2% (w/v) collagenase type I and type II mixture at 37 °C under continuous agitation. Following enzymatic digestion, the cell suspension was filtered and centrifuged, and the resulting cell pellet was resuspended in culture medium containing DMEM supplemented with 10% FBS and 1% penicillin/streptomycin. Cells were cultured in a humidified incubator at 37 °C with 5% CO_2_, and the medium was replaced every 2–3 days. Upon reaching 80–90% confluency, cells were passaged using standard trypsinization procedures. Cells at passage 4 were used for subsequent experiments.

### 2.6 In Vitro Biocompatibility and Cytotoxicity Evaluation

The cytotoxicity of the developed materials was evaluated using both direct and indirect approaches to assess cell–material interactions as well as any potential release of toxic substances. For the direct method, materials were sterilized by incubation in 70% ethanol for 20 min at room temperature, rinsed twice with PBS, and placed in a 12‐well culture plate. Human bone marrow mesenchymal stem cells (hBMSCs), cultured in complete DMEM containing 10% FBS and 1% penicillin/streptomycin, were directly seeded onto the center of each material at a density of 20,000 cells per well. First, a 5 µL droplet of the cell suspension was seeded at the center of the specimen, directly on the coated–uncoated interface, so that it was centered at the boundary in the half‐coated design, and after 30 min, an additional 20 µL was added to promote localized cell attachment. The plate was then incubated at 37 °C for 3 h. Once cell attachment was confirmed, 1 mL of medium was gently added to each well to cover the surface. Following 24 h of incubation, cell viability and attachment were examined using Calcein AM staining (2 µM; 490/520 nm). To further evaluate spatial cell distribution between coated and uncoated regions, samples were fixed with 4% paraformaldehyde (PFA) and stained with DAPI (2 nM, 45 min; excitation/emission: 350/450 nm) prior to fluorescence imaging.

For indirect cytotoxicity assessment, materials were sterilized by autoclaving and then incubated in 10 mL of complete culture medium at 37 °C for 24 h to generate material‐conditioned medium. Separately, the hBMSCs were seeded into 96‐well plates at 10,000 cells per well and allowed to adhere for 24 h. The culture medium was then replaced with the conditioned medium, and cells were incubated for an additional 24 h. Cell viability was quantified using the Cell counting kit‐8 (CCK‐8) assay, with absorbance measured at 450 nm. To assess potential morphological changes, cells were fixed with 4% PFA and stained with phalloidin (495/518 nm) for cytoskeletal visualization and DAPI (350/450 nm) for nuclear staining. Fluorescence images were compared with those of untreated control cells to determine whether the material extracts induced any structural or morphological alterations.

Two sterilization methods were intentionally used because the biological evaluation comprised direct‐contact and indirect assays, which impose different constraints and potential artifacts. For the direct‐contact test, sterilization with 70% ethanol followed by PBS rinsing was selected as a mild, low‐temperature approach that minimizes alteration of the coated/uncoated surface chemistry and interface, which is the actual substrate encountered by cells during attachment and early spreading. In contrast, for the indirect cytotoxicity (extract) assay, autoclaving was used as a robust terminal sterilization step appropriate for extract preparation, ensuring sterility of the bulk specimen while avoiding introduction of solvent‐related residues into the leaching medium.

### 2.7 Biofilm Formation on the Implants

Biofilm formation on the implant surface was assessed to evaluate the influence of coating parameters on bacterial attachment and biofilm development. For biofilm formation, an overnight culture of *Staphylococcus aureus* (*S. aureus*) EDCC 5055 (approximately 1 × 10^9^ CFU/mL) was diluted 1:100 in fresh BHI medium, resulting in an initial inoculum of approximately 1 × 10^7^ CFU/mL. Specimens measuring 10 mm × 10 mm × 5 mm were fully immersed, ensuring that all surfaces were exposed to the medium during the extraction process. *S. aureus* EDCC 5055, a commonly used biofilm‐forming strain in implant‐associated infection models, was grown by inoculating a single colony from an LB agar plate into 10 mL of BHI broth and incubating overnight at 37 °C with shaking (180 rpm). Sterile implants were then placed in an autoclaved 300 mL wide‐neck Erlenmeyer flask containing 50 mL of BHI broth, inoculated with the overnight S. aureus culture at a 1:100 ratio, mixed for 30 s, covered, and incubated under static conditions at 37 °C for 24 h; the medium was replaced every 24 h for a total of three days. On the third day, after exchanging the medium and incubating for 30 min, implants were washed twice with PBS to remove planktonic bacteria, transferred into sterile 5 mL Eppendorf tubes containing 2 mL fresh PBS, and sonicated using a BactoSonic 14.2 at 80% power for 3 min to detach the biofilm. The resulting suspensions were serially diluted (1:10), plated on LB agar using sterile single‐use spreaders, incubated for 24 h at 37 °C, and colony‐forming units (CFUs) were counted and normalized to the total PBS volume used for sonication.

### 2.8 Statistical Analysis

All experimental results, including mechanical measurements, micro‐CT‐derived parameters (e.g., porosity), and biological outcomes (cell adhesion, viability, proliferation, and antibacterial response), are reported as mean ± standard deviation (SD) based on three independent replicates per condition (n ≥ 3), unless otherwise stated. Surface roughness measurements were performed using four independent measurements per sample group (n = 4). Quantitative comparisons were evaluated using standard statistical criteria, with p < 0.05 considered statistically significant for micro‐CT and bioassay datasets. As the study primarily emphasizes process–surface–coating characterization and biological response trends rather than hypothesis‐driven group comparisons.

Where applicable, statistical comparisons were performed using one‐way ANOVA with Tukey's post‐hoc test (Figure 6) and Welch's t‐test with Holm correction for biofilm datasets (Figure 9), with p < 0.05 considered statistically significant.

## 3 Results and Discussion

Although multiple etching durations were initially screened, all subsequent analyses were focused on the optimized windows of 6 h (360 min) and 12 h (720 min), selected based on surface integrity and hierarchical roughness development. The 6 h condition is presented in the main manuscript, while 12 h (720 min), 18 h (1080 min) and 24 h (1440 min) FESEM images are provided in the Supplementary Information Figures S3–S5; the shorter etching times (e.g., 5–60 min) showed negligible differences in surface porosity/roughness. Acid etching was conducted for 5, 10, 30, 60, 360, 720, 1080, and 1440 min. Etching durations shorter than 360 min did not produce sufficiently discernible surface roughness under SEM, whereas extended etching beyond 720 min led to the onset of surface agglomeration. Therefore, 360 and 720 min were selected as the etching windows for Ti64 samples for subsequent evaluation.

### 3.1 Microstructure and Morphology of SLM‐fabricated Ti64

All SLM‐fabricated Ti64 specimens were sequentially polished to achieve a mirror finish before coating. As shown in Figure 2a–d, the surface became uniformly smooth and featureless, confirming the removal of partially melted particles, scan traces, and surface irregularities generated during selective laser melting. The inset in Figure 2a displays the cubic sample geometry used for surface analyses and coating deposition. After polishing, the samples were etched with an HNO_3_/HF solution to introduce micro‐ and nanoscale roughness. The post‐etched morphology (Figure 2e–j) exhibits fine pits and valleys formed by the preferential dissolution of the α + β phases, thereby increasing the surface area, which is favorable for coating adhesion and bioactivity. This controlled roughening marks the initial stage of surface activation, with further variations in etching time producing distinct topographical features discussed in the following section.

**FIGURE 2:**
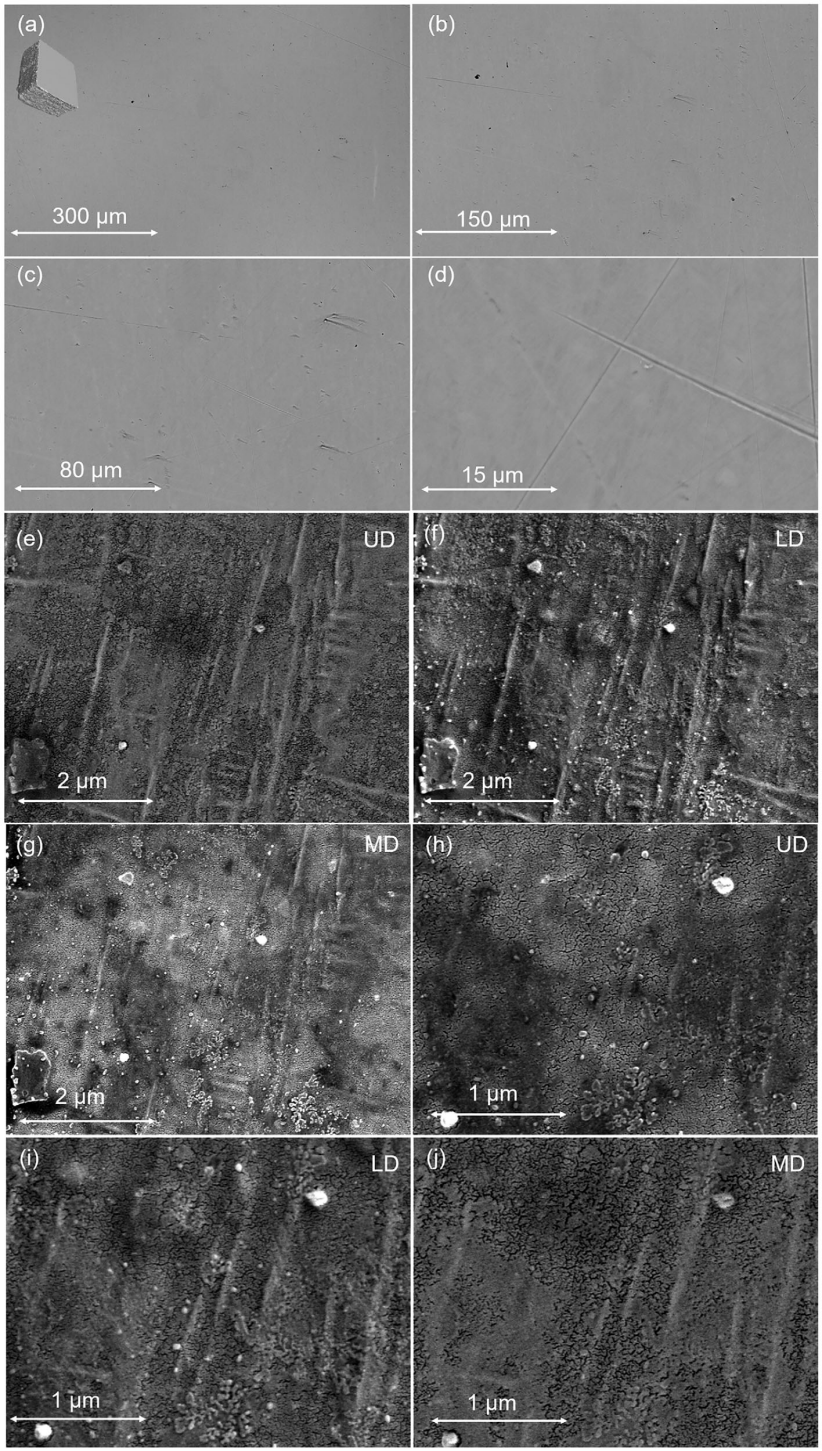
Surface preparation and initial etching morphology of SLM Ti64 samples. (a–d) Mirror‐polished surfaces showing smooth, defect‐free morphology; inset in (a) shows sample geometry. (e–j) FESEM micrographs of Ti–6Al–4 V samples etched for 6 h using an HNO_3_ + HF solution. Images (e–g) obtained with LD, MD, and UD detectors at high magnification display uniformly distributed etching pits and dissolution zones across the surface. Higher‐magnification views highlight the progressive development of micro–nano grooves and partially dissolved regions, while the highest‐resolution images (h–j) reveal a dense, interconnected nanoscale texture. The complementary detector modes emphasize topographical and compositional contrast, confirming the formation of a hierarchically roughened surface favorable for coating adhesion and biological activity.

### 3.2 Microstructural Insights into Etched SLM Surfaces

The FESEM micrographs in Figure 2e–j provide a detailed representation of the surface evolution of SLM Ti64 after 6 h of chemical etching. With increased magnification (Figure 2e–g), the development of fine dissolution grooves and sub‐micron undulations becomes evident, associated with the preferential dissolution of the β‐phase and the α/β interphase. At the nanoscale (Figure 2h–j), the surface exhibits dense nodular features and interconnected ridges, signifying complete activation of the metallic surface and an optimal roughness gradient for subsequent coating deposition. These features can be attributed to the combined influence of the initial SLM surface morphology and the chemical etching process. The as‐built SLM surface contains partially melted powder particles, melt‐pool boundaries, local pores, and defect‐prone regions generated by nonuniform melting and rapid solidification. During etching, HF removes the stable TiO_2_ layer and promotes localized dissolution of Ti‐rich regions, whereas HNO_3_ contributes to oxidation and passivation. With prolonged etching, preferential attack occurs at high‐energy sites such as particle boundaries, melt‐pool edges, pores, and weakly bonded surface particles. Consequently, pre‐existing SLM‐induced surface irregularities become exposed and enlarged, producing the defect‐like morphology observed after 6 h of etching.

A comparative examination of longer etching durations indicated that extending the treatment to 12 h (Supplementary Information Figure S3) resulted in a slightly more pronounced yet uniform micro–nano texture, preserving the structural integrity of the surface. Both 6 h and 12 h treatments thus produced hierarchically organized morphologies that balance chemical activation with surface stability, making them ideal substrates for electrophoretic deposition of HAp/rGO coatings. However, further prolongation beyond 12 h led to excessive dissolution and localized oxide formation, generating porous and irregular surface domains detrimental to coating uniformity and biological response (Supplementary Information Figure S4). Consequently, the etching window of 6–12 h is identified as the optimal regime, ensuring sufficient topographical roughness for strong coating adhesion and promoting favorable cellular interaction and osseointegration potential.

### 3.3 Microstructural Insights into HAp/rGO‐coated SLM Surfaces

Figure 3a–e presents a comprehensive multi‐scale FESEM analysis demonstrating the selective EPD of the rGO–HAp coating on the metallic substrate. At the lowest magnification in Figure 3a, the SEM image clearly reveals a distinct boundary separating the coated and uncoated halves of the specimen. This contrast arises from the markedly different secondary electron emission behavior between the two surfaces, the coated region exhibits a darker and more heterogeneous texture due to the presence of rGO–HAp particulates and increased microscale roughness, whereas the uncoated region appears smoother and brighter, consistent with a polished and minimally altered metallic surface (Figure 3 shows the results of the sample without any etching to distinguish both regions clearly). The abrupt transition between the two halves confirms that the EPD process confined deposition strictly to the intended area without observable overspray or unintended particulate migration.

**FIGURE 3:**
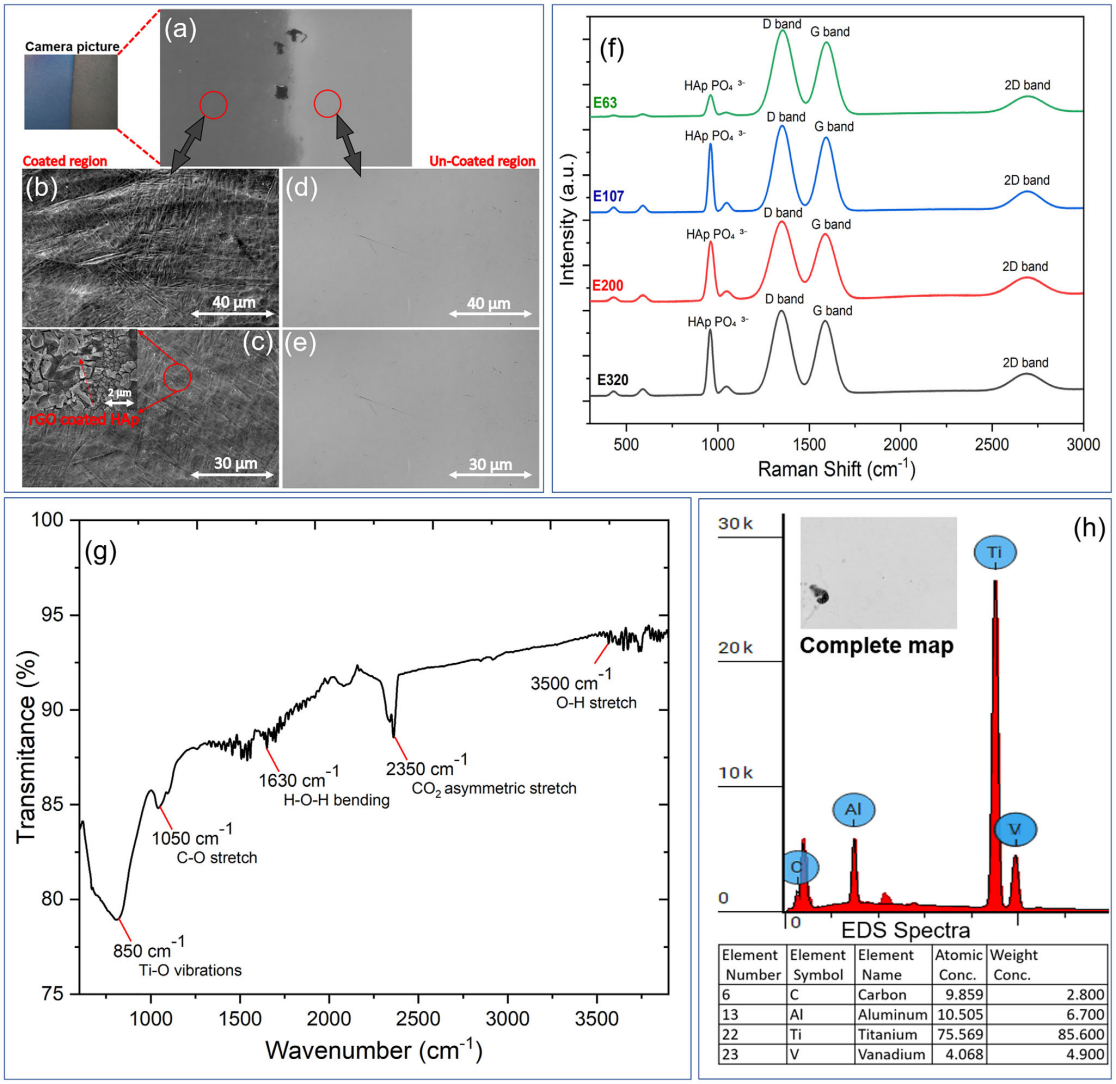
FESEM images showing selective EPD of rGO–HAp coating. (a) Low‐magnification SEM image showing distinct coated (left) and uncoated (right) surfaces. (b, c) Higher magnification SEM images of the coated region displaying surface roughness and rGO‐coated HAp clusters (inset). (d, e) SEM images of the uncoated region, showing morphology and absence of coating. (f) FTIR spectrum of the pristine Ti64 substrate showing characteristic Ti–O lattice vibrations (≈850 cm^−^^1^), adsorbed carbonate species (≈1050 and 2350 cm^−^^1^), and hydroxyl‐related bands associated with surface hydration (≈1630 and 3500 cm^−^^1^). (g) EDS spectrum and elemental map of the same pristine sample, indicating dominant Ti peaks with contributions from Al and V, consistent with the base alloy composition, and minor carbon attributed to environmental adsorption. (Supplementary Information Figure S12).

Figure 3b,c depicts SEM micrographs at higher magnifications of the coated surface. The morphology in Figure 3b displays a dense and directionally varied roughness profile, reflecting both the underlying mechanical polishing marks and the overlay of electrophoretically deposited material. This topography is a typical characteristic of HAp‐based coatings formed under relatively low‐energy deposition conditions, in which particles accumulate preferentially along surface discontinuities and ridges. Figure 3c further resolves the microstructural features and reveals the presence of granular HAp clusters embedded within or partially coated by thin, sheet‐like rGO domains. The inset in Figure 3c provides high‐resolution confirmation of the composite morphology, showing the faceted and polygonal nature of HAp particles alongside the wrinkled textures typical of rGO. The integration of these two materials is crucial for validating that the deposition successfully produced a heterogeneous bioactive layer rather than isolated particle adhesion.

In contrast, Figure 3d,e depicts the uncoated region in the specimen at magnifications equivalent to those used for Figure 3b,c. These surfaces are smooth and devoid of particulate matter, exhibiting only the polishing‐induced linear scratches and minor surface defects characteristic of the base alloy after standard metallographic preparation. The absence of micro‐ or nano‐scale features associated with rGO–HAp deposition confirms that no coating inadvertently formed on this half of the sample. Furthermore, the uniformity of the uncoated region across magnifications indicates that the sample preparation, masking strategy, and EPD parameters effectively prevented stray deposition or particle bridging at the coating boundary.

### 3.4 Elemental Mapping of Pristine Specimen

The pristine Ti64 samples were first examined using FTIR to evaluate their baseline surface chemistry prior to coating deposition. FTIR was intentionally performed only on the uncoated substrates because the infrared beam penetrates ∼1–5 µm into the material [26], depending on surface roughness, oxide thickness, and refractive index. This relatively large penetration depth means FTIR primarily detects bulk and subsurface chemical bonds, rather than the outermost atomic layers where the coating will later be deposited. As reported in the literature, FTIR cannot isolate the chemistry of nanoscale surface coatings on titanium alloys because it averages signals from both the oxide layer and the underlying metallic substrate, making it unsuitable for confirming thin‐film deposition or surface‐specific bonding chemistry [27]. The FTIR spectrum of the pristine samples, as shown in Figure 3g, shows characteristic vibrational bands corresponding to native titanium oxide (TiO_2_) and common adsorbed species. The feature at ∼850 cm^−^^1^ is attributed to Ti–O lattice vibrations, confirming the presence of the naturally formed TiO_2_ passive layer typical for Ti64 alloys. Broad absorption near ∼3500 cm^−^^1^ corresponds to O─H stretching, while the band at ∼1630 cm^−^^1^ originates from H─O─H bending, both indicative of physisorbed moisture on the oxide surface, an expected behavior for hydrophilic, freshly polished TiO_2_ surfaces. The peaks at 1050 cm^−^^1^ (C─O stretching) and 2350 cm^−^^1^ (CO_2_ asymmetric stretching) arise from atmospheric carbonates/CO_2_ adsorbed on the oxide layer, a phenomenon that has been frequently reported for SLM‐fabricated Ti–6Al–4V surfaces due to their high surface reactivity and native oxide chemistry [28].

To complement the FTIR findings, EDS was performed to quantify the elemental composition of the pristine samples, as given in Figure 3h. The spectra reveal dominant titanium peaks, along with the expected alloying elements, aluminum and vanadium, consistent with the Ti64 grade. The presence of small carbon signals is attributed to surface adsorption during handling or from environmental exposure and is commonly observed in SLM‐fabricated titanium alloys. Importantly, no foreign elements or contaminants were detected, supporting the FTIR observation that the surface is chemically clean aside from the naturally occurring oxide and environmental adsorbates. Together, the FTIR and EDS analyses confirm that the baseline condition of the SLM Ti64 substrate is governed by its inherent TiO_2_ layer and alloy composition. This establishes a necessary reference point for later XPS analysis, which is applied after coating deposition specifically because its shallow probing depth (∼5–10 nm) allows accurate identification of surface‐specific chemical states (Supplementary Information Table ST2 for detailed mapping).

### 3.5 Elemental Analysis of rGO/HAp Coated Ti64 Specimen

Raman spectra confirmed the successful incorporation of rGO into the HAp/rGO coating as given in Figure 3f. The HAp‐related phosphate vibration was observed around 960 cm^−^^1^, corresponding to the ν_1_ PO_4_^3^^−^ mode. In addition, two prominent carbon‐related bands were detected at approximately 1348–1351 cm^−^^1^ and 1586–1588 cm^−^^1^, assigned to the D and G bands of rGO, respectively. The D band is associated with defect‐induced vibrations and disordered carbon, whereas the G band corresponds to the in‐plane vibration of sp^2^‐bonded carbon atoms. The I_D_/I_G_ ratio was calculated from the integrated fitted peak areas of the D and G bands after baseline correction. The calculated ID/IG ratios were 1.21, 1.18, 1.19, and 1.15 for the HAp/rGO‐coated E63, E107, E200, and E320 samples, respectively. These values indicate the defect‐rich nature of rGO within the composite coating across all VED conditions. The comparable ID/IG ratios suggest that rGO was successfully incorporated in all coated samples, while the small differences may be related to local variations in coating distribution and rGO structural disorder on the differently processed Ti64 surfaces. Table 2 presents representative Raman peak parameters used to illustrate the calculation of the I_D_/I_G_ ratio.

**TABLE 2:** Raman peak parameters of HAp/rGO coatings on E63, E107, E200, and E320 Ti64 samples, showing the HAp phosphate vibration around 960 cm^−^^1^ and the characteristic rGO D and G bands around 1350 and 1580 cm^−^^1^, respectively.

Sample	D‐band position (cm^−^^1^)	G‐band position (cm^−^^1^)	D‐band fitted area, (A_D_)	G‐band fitted area, (A_G_)	(I_D_/I_G_ = A_D_/A_G_)
HAp/rGO coating E63	1348	1586	12850	10600	1.21
HAp/rGO coating E107	1349	1587	12510	10600	1.18
HAp/rGO coating E200	1348	1587	12555	10550	1.19
HAp/rGO coating E320	1351	1588	12120	10540	1.15

A survey of the literature shows that although rGO/HAp composite powders and bulk materials have been analyzed extensively by XPS, similar analyses of coatings are almost entirely absent. Nosrati et al., [29] reported one of the most detailed XPS characterizations for rGO/HAp systems, but their work focused exclusively on bulk nanocomposites consolidated by spark plasma sintering rather than coatings on metallic substrates. In contrast, several studies have investigated hydroxyapatite coatings combined with graphene or graphene oxide on titanium surfaces [30]. Janković et al. [31] deposited the HAp/graphene composite films onto commercially pure titanium and used XPS to confirm the presence of graphitic carbon, though their system did not contain rGO and was not applied to Ti64. Bulbul et al. examined HAp/rGO coatings on laser‐treated Ti–6Al–4 V and reported structural, mechanical and corrosion behavior without carrying out XPS [14]. Therefore, the present work addresses this gap by providing a comprehensive XPS chemical‐state analysis of rGO/HAp coatings on Ti64, offering insight into the bonding environments, surface chemistry, and interface characteristics of this hybrid nanocomposite system.

The survey spectrum presented in Figure 4a reveals the chemical signature expected from a rGO/HAp composite deposited on a Ti64 substrate. The presence of strong oxygen and carbon peaks indicates that the surface is dominated by a carbon/oxygen‐rich overlayer consistent with rGO sheets mixed with calcium phosphate. The Ti signal remains detectable indicating a relatively thin overlayer within the XPS sampling depth. This partial visibility is typical for thin electrophoretically deposited HAp/rGO films on Ti64, where the attenuation length of the photoelectrons allows Ti 2p peaks to bleed through unless the coating exceeds several tens of nanometers [29]. The clear Ca 2p and P 2p contributions verify that HAp occupies the near‐surface region accessible to XPS, implying that the coating presents a mixed chemical interface in which both rGO and HAp are surface‐active.

**FIGURE 4:**
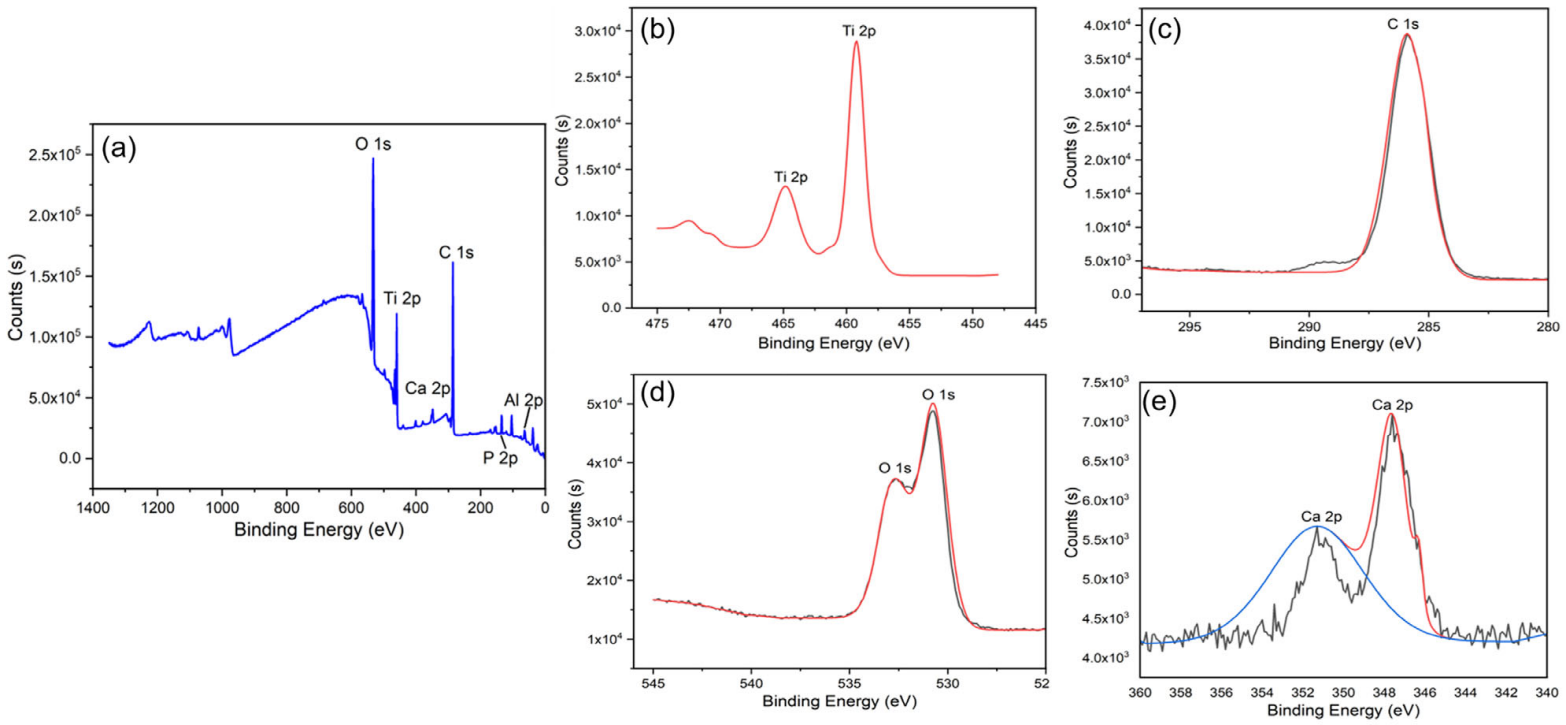
(a) XPS analysis of the rGO/HAp‐coated Ti64 samples. Survey spectrum showing the presence of C, O, Ca, P, and Ti corresponding to the rGO/HAp composite layer and the underlying Ti64 substrate. (b) Ti 2p spectrum corresponding to oxidized titanium from the TiO_2_‐enriched native oxide layer, with the absence of metallic Ti confirming complete coverage by the composite coating. (c) High‐resolution C 1s spectrum dominated by the graphitic C─C component of reduced graphene oxide with residual C─O/C═O functionalities. (d) O 1s spectrum containing lattice oxygen from TiO_2_ and PO_4_^3^^−^ groups together with higher‐binding‐energy oxygen species associated with hydroxyl and carbon–oxygen groups from rGO and surface hydroxylation. (e) Ca 2p doublet characteristic of Ca^2^^+^ in hydroxyapatite, with slight broadening attributed to nanostructured HAp and interfacial interactions with oxygenated groups on rGO.

The Ti 2p core‐level spectrum in Figure 4b shows two peaks at positions characteristic of oxidized titanium: Ti 2p3/2 at 458.5 eV and Ti 2p1/2 at 464.2 eV. The absence of metallic titanium signals confirms that the surface titanium from the alloy is covered by a stable oxide film, likely the native TiO_2_‐enriched layer that forms on Ti64 during all wet and thermal processes [32]. Although Ti 2p remains detectable, the absence of a metallic Ti component and the dominance of Ti–O states suggest that the near‐surface titanium is predominantly oxidized, while the coating is thin enough that underlying Ti–O/substrate contributions are still sensed by XPS; this is consistent with HAp/rGO coatings, which usually exhibit mixed surface coverage, nanoscale porosity, and partial transparency to X‐rays [33].

The C 1s spectrum in Figure 4c is dominated by a single asymmetric peak centered near 284.6–284.8 eV, corresponding to sp^2^/sp^3^ carbon bonds from reduced graphene oxide. The tail toward higher binding energy suggests the presence of residual oxygenated carbon species, mainly C–O or C = O functionalities, which are typical of partially reduced graphene oxide [34]. The persistence of a weak shoulder above 286 eV indicates that some carboxyl or hydroxyl functional groups remain [34, 35]. In rGO/HAp composite coatings, this is expected because calcium ions from hydroxyapatite often interact electrostatically with residual carboxyl groups on rGO, stabilizing them and preventing complete reduction [36]. This behavior is consistent with several literature reports on rGO/HAp powders and sol–gel composites, where carboxyl peaks persist due to Ca^2^^+^ bonding [36]. The features observed in the C 1s spectrum therefore suggest that the graphene component is partially reduced, while retaining sufficient oxygenated functionalities to enable chemical interactions and interfacial coupling with the hydroxyapatite phase.

The O 1s in Figure 4d spectrum shows two distinct components. The lower‐binding‐energy peak near 530.0–530.5 eV corresponds to lattice oxygen in TiO_2_ as well as the phosphate oxygen in the PO_4_^3^^−^ groups of hydroxyapatite. The second higher‐binding‐energy peak around 531.5–532.5 eV corresponds to oxygen in hydroxyl, carbonyl, and epoxy groups, arising from the rGO sheets and possibly from surface hydroxylation of TiO_2_. The relative intensity of the higher‐energy oxygen peak suggests that rGO is present at or near the surface and contributes significantly to oxygen chemistry. The coexistence of both oxygen environments confirms that the surface region contains both rGO and hydroxyapatite and that the coating is chemically mixed rather than forming isolated domains [37, 38].

The Ca 2p in Figure 4e region presents a clear doublet corresponding to Ca 2p3/2 and Ca 2p1/2, typically observed around 347.0–347.5 eV and 350.5–351.0 eV for stoichiometric hydroxyapatite. The observed peak positions fall very close to these values, indicating that the calcium phosphate phase has not decomposed into secondary phases such as CaO or CaCO_3_ during deposition or drying. The line shape appears broadened relative to dense crystalline HAp, which is common for electrophoretically deposited HAp coatings where the calcium phosphate is nanoscale, partially amorphous, or interacts with rGO sheets. The broadening on the lower‐energy side may also reflect interfacial environments in which Ca^2^^+^ interacts with carboxyl groups on rGO, consistent with the behavior observed in powder systems by Nosrati et al. [29] and others [39].

The complete spectra reveal a surface composed of partially reduced graphene oxide intertwined with nanoscale hydroxyapatite, deposited as a relatively thin layer on Ti64, such that the substrate oxide remains detectable. The carbon and oxygen spectra show the chemical signatures of reduced graphene oxide with residual functional groups, while the Ca 2p and P 2p peaks confirm the chemical integrity of the hydroxyapatite phase. The simultaneous visibility of Ti 2p suggests a coating thickness below the full attenuation length of XPS electrons, or a coating with micro‐ or nano‐scale porosity allowing some substrate contribution. An important observation from the XPS analysis is the absence of a detectable vanadium signal from the coated Ti64 samples. This can be attributed to the combined effects of the low V content in Ti64, attenuation by the HAp/rGO coating and surface oxide layer, and the surface‐sensitive nature of XPS. Although the Ti/TiO_2_‐related signal remained detectable due to the high Ti content of the substrate and the dominance of Ti‐based surface oxides, the weaker V signal may have been attenuated below the XPS detection limit. Similar behavior has been reported for as‐fabricated SLM Ti64, where V was not detected by XPS despite its presence in the alloy, indicating that V detection is strongly influenced by surface oxide chemistry, surface morphology, and sampling depth [40]. Vanadium ions are known to exhibit cytotoxic and pro‐inflammatory effects when released from Ti64 surfaces, and their suppression is therefore desirable for long‐term biocompatibility [41]. However, the absence of a detectable V signal in the present XPS spectra should be interpreted only as evidence of surface‐level signal attenuation within the XPS sampling depth. It does not provide direct evidence of reduced V ion release under physiological conditions. Therefore, direct evaluation of V release would require dedicated elution, corrosion‐assisted release, or leaching assays, such as ICP‐MS, which were beyond the scope of the present surface‐chemistry analysis. Such shielding could contribute to improved biological safety, reduced local tissue irritation, and potentially more favorable healing responses following implantation (Supplementary files for XPS graphs of second sample Figure S13 and Table TS3 for detailed peaks mapping).

### 3.6 Micro‐computed Tomography (Micro‐CT) Analysis of Ti64 Samples

Figure 5 presents the three‐dimensional pore architecture and quantitative defect data for the Ti64 samples produced under different VEDs. The measured porosity decreases consistently with increasing energy input, confirming that melt‐pool stability and defect evolution are strongly governed by the applied VED. At 63 J/mm^3^ (E63), the total porosity reached about 1.70%, with elongated, irregular voids concentrated along track and layer boundaries [42]. The relatively large D_80_ value (≈ 0.23 mm) indicates that the pore‐volume fraction was dominated by coarse, interconnected features typical of lack‐of‐fusion (LOF) defects produced by insufficient laser penetration and incomplete bonding between adjacent melt pools. Similar morphologies have been associated with inadequate overlap and low linear energy density, which restricts remelting of the preceding layer and leaves unmolten powder zones that solidify as planar voids [43].

**FIGURE 5:**
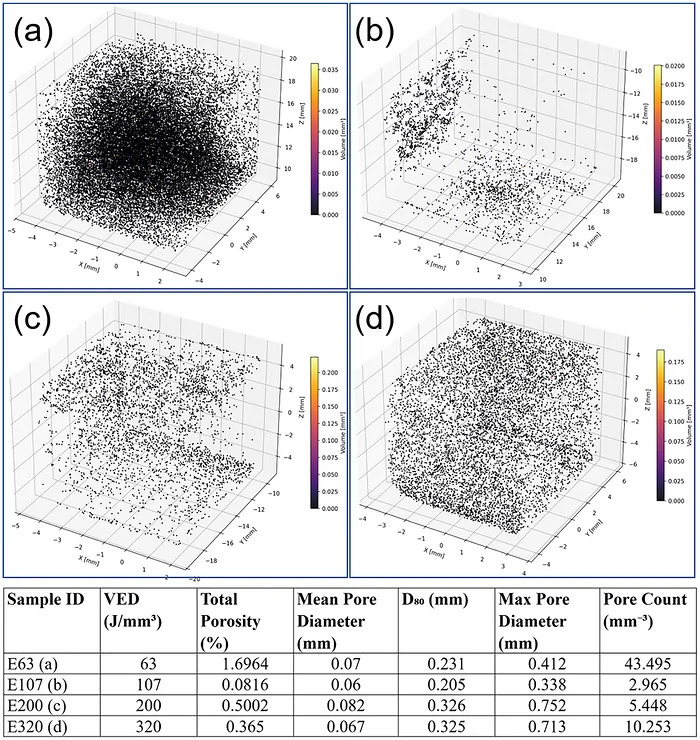
Three‐dimensional spatial distribution of pores in Ti64 samples reconstructed from micro‐CT analysis. (a) Sample E63 presents the highest pore density, with a continuous cloud‐like distribution of small‐volume pores covering the entire scanned region. (b) Sample E107 showing sparse and clustered pores, primarily located near the upper and lower regions of the scan volume. (c) Sample E200 exhibiting a more uniform distribution with an increased number of fine pores throughout the sample. (d) Sample E320 showing an increased pore population consistent with excessive energy input driving keyhole‐mode melting; vaporization‐induced instability and keyhole collapse can trap gas and generate keyhole porosity. Color bars quantify individual pore volumes, enabling differentiation between small gas pores and larger lack‐of‐fusion defects. The table summarizes total porosity, mean and maximum pore diameters, D_80_ (pore diameter at 80% cumulative pore‐volume fraction), and pore count density (N / mm^3^). Differences in pore morphology correspond to melt‐pool stability and defect formation mechanisms such as lack‐of‐fusion or keyhole porosity.

As the VED increased to 200–320 J/mm^3^, the total porosity fell to the 0.36–0.50% range, and the pores became more equiaxed and isolated. The D_80_ values (≈ 0.32–0.33 mm) suggest that larger, but fewer, gas‐type pores dominate under these conditions [43]. This shift corresponds to the transition from LOF to keyhole‐induced porosity. Higher energy promotes deeper melt pools and stronger Marangoni convection, but also increases the risk of vapor‐cavity formation and instability when the local temperature exceeds the Ti64 boiling threshold. The resulting spherical or near‐spherical pores reflect gas entrapment and collapse events within transient keyholes [44]. At the optimized 107 J/mm^3^ condition (E107), the structure is almost fully dense, with porosity limited to 0.08%. The pores are small (mean ≈ 0.06 mm, D_80_ ≈ 0.20 mm) and uniformly distributed, demonstrating a stable melt pool with complete inter‐track fusion. The fine, homogeneous pore population observed here is characteristic of balanced melting and solidification kinetics, where sufficient energy density eliminates LOF without inducing keyhole instability [44].

Figure 5a–d shows the three‐dimensional spatial distribution of pores within the Ti64 samples (E63, E107, E200, and E320) reconstructed through micro‐computed tomography. In Figure 5a, corresponding to sample E63, a dense population of irregular and interconnected pores is observed. The pores are elongated and preferentially oriented along the build and hatch directions, suggesting incomplete melting and interlayer bonding typical of lack‐of‐fusion defects generated under insufficient energy input. The relatively high defect connectivity reflects unstable melt‐pool dynamics and poor wetting between adjacent tracks [45]. In contrast, Figure 5b (E107) shows a more homogeneous pore distribution, with smaller, spherical pores that are sparsely distributed. This morphology indicates adequate energy input for full consolidation of the powder layer, resulting in reduced porosity and the elimination of unmolten zones. The uniform pore field and lower D_80_ value (0.205 mm) highlight improved melt‐pool stability at this intermediate energy condition [46].

At higher energy levels (E200 and E320; Figure 5c,d, respectively), the internal architecture transitions further toward rounded, isolated pores dispersed evenly throughout the sample. The spatial distribution suggests the predominance of gas‐entrapped or keyhole‐induced porosity, typically arising from the transient collapse of vapor cavities in deeper melt pools [47]. The D_80_ values of 0.325 and 0.326 mm correspond to slightly larger but less interconnected pores, reflecting efficient melting but occasional vapor entrapment during solidification. Across all conditions, the pore number density and size distribution follow a clear trend with increasing energy density: large, irregular, and connected pores at low energy (E63) progressively evolve into smaller, isolated, and more spherical voids at higher energies (E107–E320) [43] (Supplementary Information for Equivalent diameter and cumulative volume fraction graphs indication D80 values of all concerned samples Figures S6–S10).

### 3.7 Surface Roughness Characterization by Profilometry

Surface roughness parameters (Ra, Rq) were measured in accordance with ISO 4287/4288 using a stylus profilometer (tip radius 2.5 µm, applied load 10 mg, sampling resolution 0.167 µm/pt) as given in Figure 6. The cut‐off wavelength (λc) was selected based on the measured roughness, i.e., λc = 0.8 mm for samples with Ra < 2 µm and λc = 2.5 mm for samples with Ra > 2 µm, following ISO 4288 recommendations. For λc = 0.8 mm, five sampling lengths were used (evaluation length = 4.0 mm). For samples with Ra > 2 µm, a cut‐off wavelength of 2.5 mm was selected according to ISO 4288. Since the specimen size (10 mm × 10 mm) does not allow the full evaluation length of 12.5 mm (5 sampling lengths), the maximum possible evaluation length of ∼8.0 mm (≈3 sampling lengths) was used, as permitted by ISO 4288 for limited surface dimensions. Detailed results are given in Figure 6c. After polishing, the Ti64 surface is expected to exhibit low surface roughness and limited surface energy, which typically restricts initial cell attachment due to the absence of micro‐/nano‐scale anchoring features as given in Figure 6b. Subsequent acid etching is anticipated to introduce hierarchical micro‐ and nano‐topographical features while simultaneously increasing surface wettability and chemical reactivity, thereby promoting enhanced early cell adhesion. Building on this etched surface, the deposition of a HAp/rGO composite coating is expected to further improve cell attachment by providing a bioactive, osteoconductive interface that closely mimics the mineral phase of bone.

**FIGURE 6:**
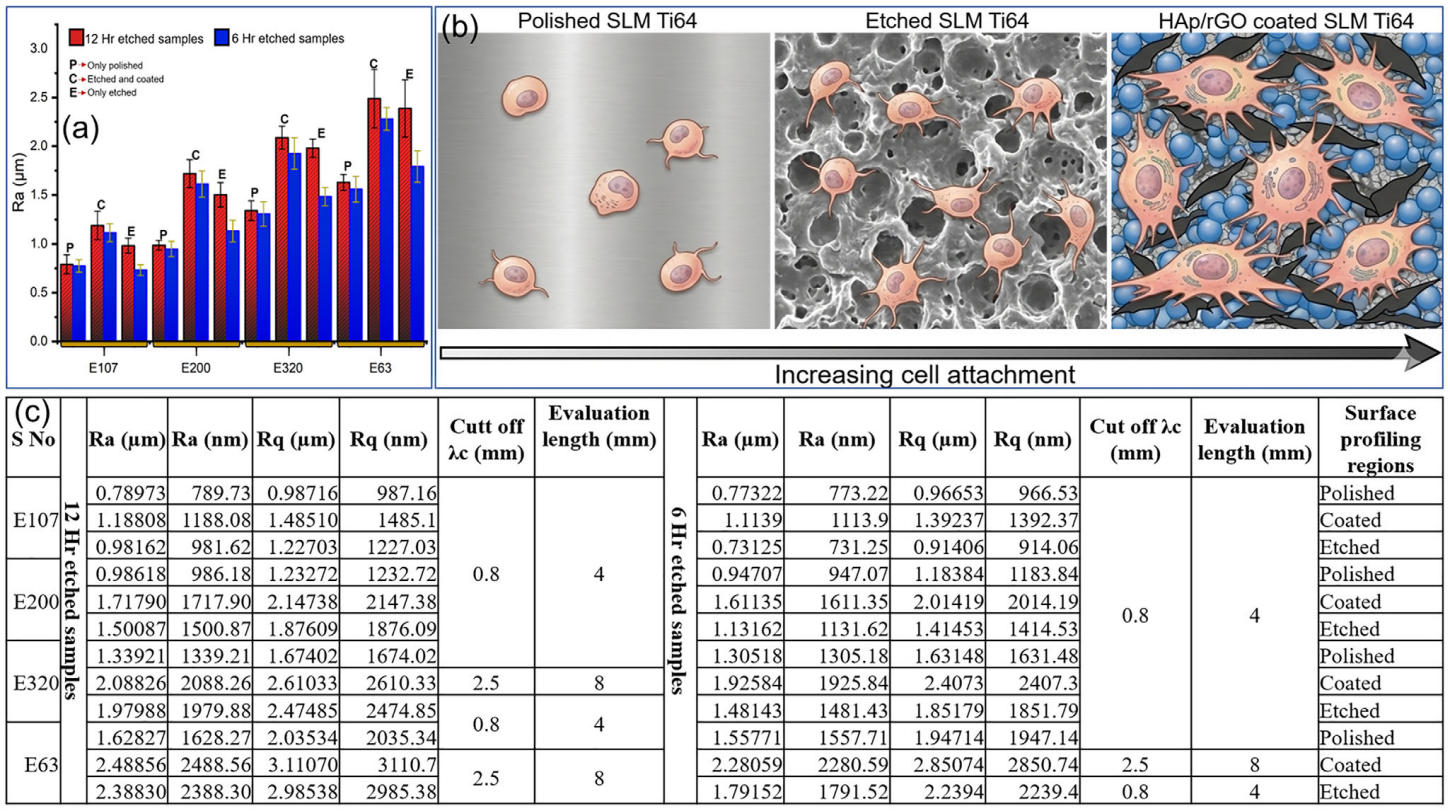
(a) Average surface roughness (Ra) of SLM‐fabricated Ti64 samples produced at different volumetric energy densities (VED: E107, E200, E320, and E63) after polishing (P), etching (E), and etching followed by rGO/HAp coating (C). Results are shown for both 6 and 12 h acid‐etched conditions. Data represent mean ± SD (n = 4). Statistical comparisons between surface conditions within each VED group were performed using one‐way ANOVA with Tukey's post‐hoc test, with significance defined at p < 0.05. (b) Schematic illustration for expected cell attachment on polished surface, etched surface and coated surface. (c) Surface roughness parameters (Ra and Rq) of SLM‐fabricated Ti64 samples for different post‐processing conditions. Data are presented as mean ± SD (n = 4). Statistical significance between conditions within each VED group was assessed using one‐way ANOVA followed by Tukey's post‐hoc test (p < 0.05).

The biological implications of these surface conditions are well established. The literature consistently shows that surface roughness is a primary determinant of how cells interact with metallic implants. Surfaces with Ra below approximately 0.5 µm provide insufficient topographical cues for osteoblasts [48], reducing adhesion strength and delaying osseointegration. Conversely, excessively rough surfaces (Ra >10–20 µm), such as as‐built AM surfaces with partially sintered powder, can impair cell viability by creating loose particles, increasing metal ion release, and decreasing wettability. Indeed, several studies have demonstrated that reducing roughness from multi‐micron to sub‐micron levels via laser polishing significantly increases cell viability, confirming that extreme surface irregularity can hinder early biological responses [48]. Within this context, the surfaces generated in the present study fall squarely within the micro‐textured regime known to promote osteogenic activity. Micro‐roughness in the range of approximately 1–5 µm Ra enhances fibrin retention, improves wettability, and enables mechanical interlocking between cells and the surface. More importantly, osteoblasts growing on moderately rough titanium activate integrin‐mediated signaling pathways that stimulate osteogenic differentiation [48, 49]. This is in contrast to smooth surfaces, where reduced roughness limits cell spreading and downregulates markers of bone formation. The presence of hierarchical features, combining etched micro‐pits with nanoscale rGO/HAp structures, further enhances biological compatibility. Such multi‐scale textures have been shown to improve protein adsorption and support enhanced cell proliferation and differentiation [50].

Across the four SLM Ti64 samples, surface roughness evolution tracks the applied VED (63–320 J/mm^3^) because VED governs density, melt pool stability, and defect/porosity distribution, which then controls how the polished surface responds to chemical etching and subsequent coating (with porosity trends in Figure 5 reflected in roughness in Figure 6a,c). E107 (107 J/mm^3^) shows the lowest porosity (0.0816%) and smallest maximum pore diameter (0.338 mm), giving the smoothest polished surface (Ra ≈ 0.78 ± 0.05 µm); with few surface‐connected pores, etching produces limited topographical development, with Ra ∼0.73 µm after 6 h and ∼0.98 µm after 12 h, and even after coating it reaches only ∼1.18 µm (lowest overall). By contrast, E63 (63 J/mm^3^) has the highest porosity (1.6964%), largest maximum pore diameter (0.412 mm), and highest pore density (43.495 pores/mm^3^), providing a much larger reactive interfacial area during etching: its polished Ra is already ∼1.62 µm, rising to ∼1.79 µm after 6 h and peaking at ∼2.38 µm after 12 h, and after coating it reaches ∼2.48 µm (highest overall), consistent with heterogeneous melt pool topology and greater defect exposure generating deeper/more numerous etch pits and a complex micro‐topography favorable for biological interactions. E200 and E320 show intermediate behavior consistent with their moderate porosities (0.5002% and 0.365%): E200 increases from ∼0.98 µm (polished) to ∼1.13 µm (6 h) and ∼1.50 µm (12 h), reaching ∼1.71 µm after coating, while E320 transitions from ∼1.32 µm to ∼1.48 µm and ∼1.97 µm after extended etching, reaching ∼2.10 µm after coating; overall, greater melt pool irregularity and pore connectivity (E63> E320> E200> E107) produce the largest roughness enhancement during chemical treatment.

The roughness progression from polished to etched to rGO/HAp‐coated surfaces is biologically advantageous: polishing provides the baseline, etching creates osteointegrative micro‐textures, and the coating adds nanostructure that enhances cell adhesion. Since etching response depends on the VED‐driven microstructure, final roughness can be tuned through both SLM parameters and post‐treatment conditions [49, 50]. These results show that SLM energy density, chemical etching duration, and nanocomposite coating can be orchestrated to achieve the optimal roughness window for cell attachment, viability, and subsequent tissue formation.

### 3.8 Adhesion Integrity of the HAp/rGO Coating

Progressive‐load scratch testing was used to evaluate the adhesion integrity of the HAp/rGO coatings on the Ti64 substrates. The coating damage developed progressively with increasing normal load and was classified into three critical stages: first cohesive cracking within the coating (L_c1_), initial coating delamination from the substrate (L_c2_), and complete coating removal of the substrate (L_c3_).

As shown in Table 3, the E107 sample exhibited the highest critical loads among all coated samples, with (L_c1_), (L_c2_), and (L_c3_) values of (8.1±1.0) N, (18.9±1.8) N, and (30.6±2.7) N, respectively. This indicates the strongest coating–substrate adhesion and better resistance to scratch‐induced coating failure. The improved adhesion of the E107 sample can be attributed to its comparatively stable melt‐pool condition and lower surface defect density, which likely provided a more uniform substrate for HAp/rGO coating deposition.

**TABLE 3:** Critical scratch loads of HAp/rGO coatings, showing cohesive cracking (L_c1_), initial delamination (L_c2_), and complete removal (L_c3_).

Sample	L_c1_ cohesive cracking (N)	L_c2_ initial delamination (N)	L_c3_ complete removal (N)
E63	(5.2 ± 0.8)	(12.6 ± 1.5)	(21.4 ± 2.1)
E107	(8.1 ± 1.0)	(18.9 ± 1.8)	(30.6 ± 2.7)
E200	(7.3 ± 0.9)	(17.2 ± 1.6)	(28.4 ± 2.4)
E320	(6.4 ± 0.7)	(15.1 ± 1.4)	(25.7 ± 2.3)

In contrast, the E63 sample showed the lowest critical loads, with (L_c1_), (L_c2_), and (L_c3_) values of (5.2±0.8) N, (12.6±1.5) N, and (21.4±2.1) N, respectively. The lower critical loads suggest weaker coating integrity and earlier coating failure under mechanical scratching. This behavior may be associated with the higher porosity and lack‐of‐fusion defects typically observed at low volumetric energy density, which can reduce coating uniformity and interfacial stability.

The E200 and E320 samples showed intermediate adhesion behavior. Although higher energy density conditions may increase surface roughness and promote mechanical interlocking, excessive energy input can also introduce local surface irregularities and defect‐related stress concentration sites, which may contribute to earlier coating failure compared with E107. (Supplementary Information for the representative progressive‐load scratch‐test tracks, magnified failure regions, and corresponding friction‐force/normal‐load curves used to identify Lc1, Lc2, and Lc3 is provided in Figure S11).

## 4 Biological Evaluation

### 4.1 Mesenchymal Stem Cell Adhesion Response to Hierarchically Engineered SLM Ti64 Surfaces

Early MSC adhesion assays are essential for evaluating the biological suitability of implant surfaces because MSC attachment, cytoskeletal tension development, and focal adhesion maturation constitute the earliest determinants of osteogenic lineage commitment and eventual osseointegration. Surface topography plays a central role in regulating the adsorption of adhesion‐relevant extracellular matrix (ECM) proteins such as fibronectin and vitronectin, whose conformation and density directly influence integrin activation and downstream mechanotransducive signaling. Numerous studies have demonstrated that micro‐ to nanoscale surface features on titanium substrates significantly enhance MSC attachment, spreading, and osteogenic differentiation, whereas excessively smooth or excessively rough surfaces impair optimal adhesion. In additively manufactured Ti64, the as‐built surface morphology and the responsiveness to subsequent etching or coating are governed by the VED, which determines melt‐pool stability, porosity formation, and microstructural roughness. Understanding how these SLM‐derived surface states influence MSC adhesion is therefore an indispensable step toward engineering biologically competent implant surfaces [51].

Figure 7a presents a two‐surface competitive adhesion assay designed to directly assess the intrinsic preference of live MSCs when simultaneously presented with two adjacent Ti64 surface states. This configuration allows MSCs to explore both surfaces under identical culture conditions, enabling a direct functional comparison of surface‐dependent adhesion and expansion behavior. The fluorescence micrograph in Calcein AM staining (Figure 7a) reveals a distinct discontinuity in cell coverage at the interface between the two surfaces. On the left region rGO/HAp coated Ti64 surface, MSCs form a dense, confluent layer with elongated morphology and aligned cytoskeletal architecture features indicative of high integrin engagement. In contrast, the right region (uncoated Ti64 surface) displays sparse MSC attachment with limited spreading, reflecting inadequate protein adsorption and insufficient nontopographical cues necessary for stable adhesion. This observation is consistent with previous findings showing that etched or nanostructured titanium surfaces significantly increase ECM protein adsorption and integrin clustering, thereby enhancing MSC adhesion and proliferation [51, 52].

**FIGURE 7:**
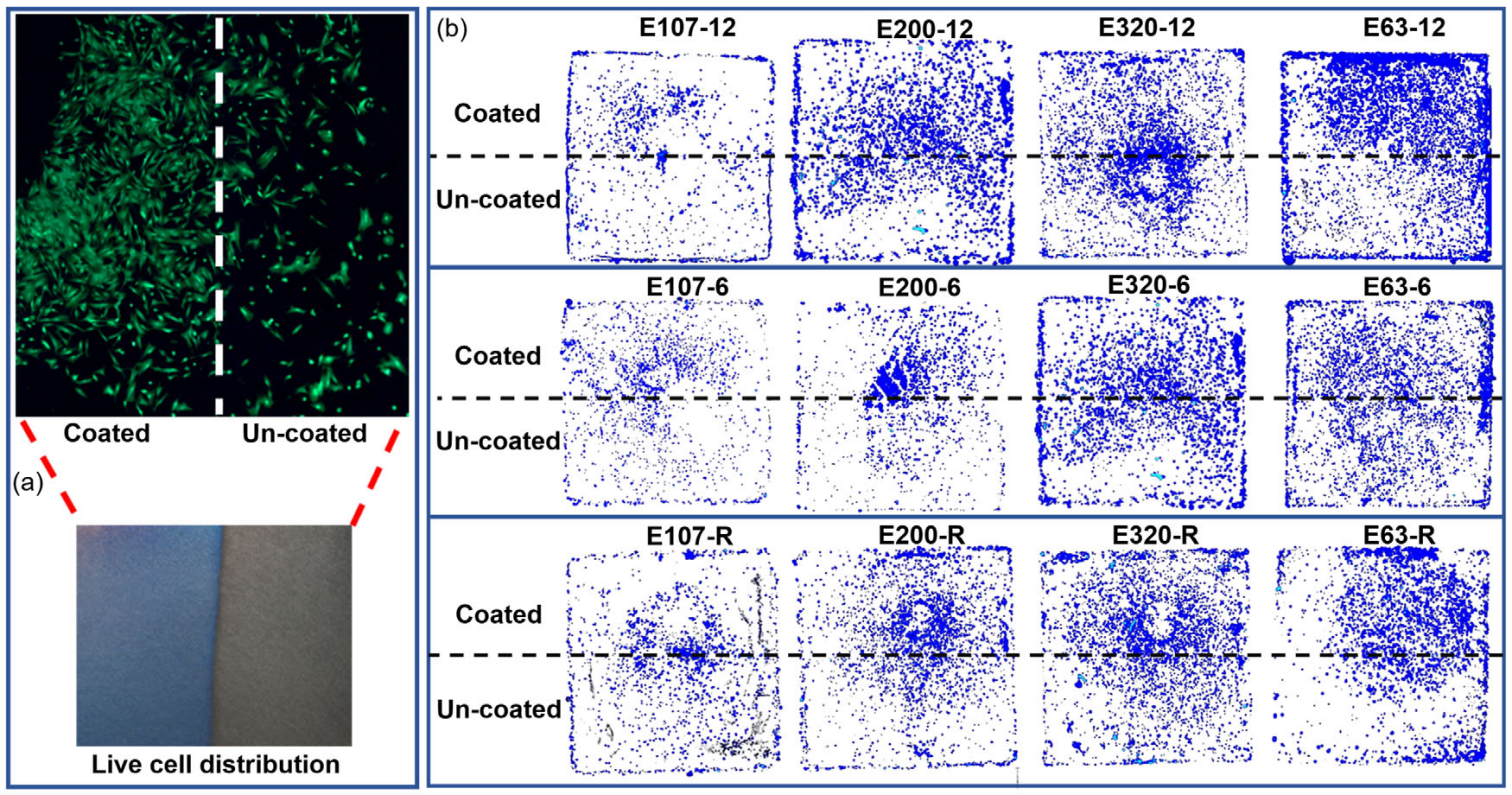
(a) Competitive MSC adhesion on two adjacent Ti64 surfaces showing strong MSC preference for the etched + rGO/HAp‐coated region, with dense and well‐spread cells, compared to sparse attachment on the uncoated smooth surface. (b) MSC attachment across SLM‐fabricated samples (E107, E200, E320, E63) after etching and coating, demonstrating highest cell coverage on hierarchically textured E63 surfaces and lowest attachment on smoother high‐VED surfaces. These results highlight the dependence of early MSC adhesion on micro/nanotopographical features.

Figure 7b extends these mechanistic insights by mapping MSC distribution across all SLM‐fabricated Ti64 specimens processed under different VED conditions (E107, E200, E320, E63) and subjected to polishing, controlled acid etching, or rGO/HAp coating. The distribution of DAPI‐stained nuclei provides a quantitative representation of early MSC attachment and spatial uniformity. A clear VED‐dependent trend emerges specimens fabricated at lower VED (particularly E63) consistently exhibit the highest MSC coverage in both etched and coated states. This can be attributed to the inherent microstructural characteristics of low‐VED SLM, which include pronounced melt‐pool boundary roughness, localized porosity, and enhanced surface reactivity features that render the surface highly susceptible to etching and capable of generating well‐defined micro‐pits and hierarchical textures [53]. Similar behavior has been reported in the literature, where controlled micro‐roughness in the 1–2 µm range enhances osteoblast and MSC adhesion by promoting ECM adsorption and focal‐adhesion assembly. The etched + rGO/Hap‐coated E63 surfaces in Figure 7b, therefore, represent the optimal synergy between SLM‐derived microtopography, acid‐etch‐enhanced microscale roughness, and coating‐induced nanoscale features, resulting in dense, uniform, and well‐organized MSC colonization [53]. The rGO/HAp layer consistently enhances MSC attachment relative to only‐etched surfaces, illustrating the importance of nanoscale biochemical and topographical features in complementing microscale roughness. This hierarchical micro–nano architecture is known to synergistically promote MSC adhesion, proliferation, and osteogenic gene expression by combining mechanical anchoring sites with bioactive chemical cues [54].

### 4.2 In Vitro Cytocompatibility Evaluation of Ti‐64 Surfaces

The cell viability assay was conducted on the entire implant to assess potential release of toxic substances, and the results (Figure 8a; Supplementary materials file Table ST1) demonstrate that all SLM‐fabricated Ti64 surfaces,‐whether pristine, etched (6 or 12 h), and etched +rGO/HAp coating‐ exhibited excellent cytocompatibility toward MSCs over both 24 hand 48 h. Cell viability results were quantitatively interpreted using ISO 10993‐5 criteria. ISO 10993‐5 considers a material/extract cytotoxic when it reduces cell viability by more than 30% relative to the negative control (i.e., viability < 70%) in quantitative assays. In the present study, all Ti64 surface conditions (polished, etched, and HAp/rGO‐coated) maintained viability > 90%, well above the ISO cytotoxicity threshold, indicating non‐cytotoxic behavior and excellent cytocompatibility under the tested conditions. No significant toxicity was observed in the cells treated with implant‐conditioned media compared to the untreated group over 48 h, indicating that no toxic compounds were released from the coated area during this time. The distribution of these values is reflected in the viability plot of Figure 8a, indicating consistent metabolic activity irrespective of VED group or surface modification. Notably, pristine E107, E63, E200, and E320 samples all demonstrated viability above 93%, confirming that unmodified SLM surfaces already satisfy cytocompatibility expectations.

**FIGURE 8:**
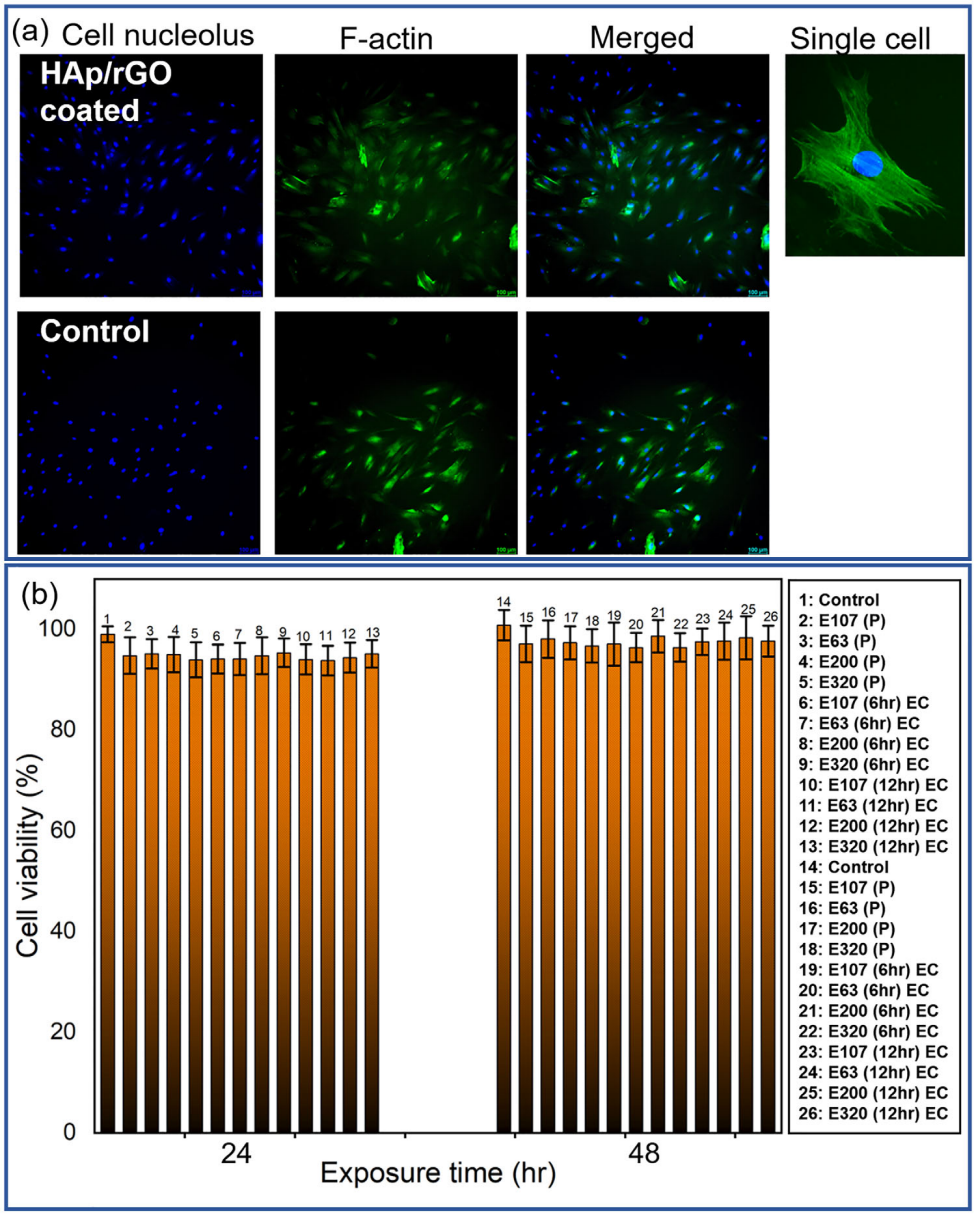
(a) Fluorescence images showing MSC adhesion, spreading, and cytoskeletal organization upon exposure to the condition media of the coated Ti64 surface after 24 h of culture. DAPI staining (blue) illustrates uniform nuclear distribution with healthy nuclear morphology, while FITC‐phalloidin staining (green) demonstrates extensive F‐actin polymerization, elongated spindle‐shaped cells, and well‐developed stress fibers. The magnified image highlights a single MSC exhibiting broad lamellipodial extensions and robust cytoskeletal structure, confirming excellent cellular health in morphology. Scale bar: 100 µm. (b) Cell viability (%) of MSCs cultured with the conditioned media exposed to SLM‐fabricated Ti64 samples from four VED groups (E107, E200, E320, E63) under pristine, etched‐6 h+ coated, and etched‐12 h+ coated conditions after 24 and 48 h of exposure. Each point represents the mean viability value for a given sample treatment combination, with red error bars indicating standard deviation (SD). Control groups at both timepoints show baseline MSC viability in the absence of materials. All sample groups maintain viability >90%, indicating no significant cytotoxic response to any surface condition. A slight decrease was observed after 24 h, followed by an increase in viability after 48 h, indicating that the cells adapted to the new environment.

The etched‐and‐coated groups maintained viability values equivalent to those of pristine specimens, with no indication of cytotoxicity despite the introduction of micro‐ and nanotopographical features and bioactive coating materials. This is consistent with the literature showing that acid‐etched titanium and calcium‐phosphate‐based nanocomposites do not impair MSC metabolic activity at early timepoints [53]. After 48 h, the viability trend, regardless of VED group or surface modification, was 96.3–98.6%. The slight increase relative to the 24 h values reflects normal MSC proliferation rather than any surface‐dependent effect. The evidence from Figure 8a demonstrates that none of the engineered surfaces induce cytotoxicity. All viability values consistently exceed 93%, and by 48 h approach or surpass those of the control. These findings confirm that the SLM‐derived microstructures, the subsequent etching processes, and the rGO/HAp coating strategy are all fully compatible with MSC survival and early proliferation.

### 4.3 Fluorescence Imaging of MSC Adhesion and Cytoskeletal Organization

Fluorescence staining of MSCs cultured with the conditioned media of the HAp/rGO‐coated Ti64 sample demonstrated excellent cellular health, with strong evidence of robust attachment, spreading, and cytoskeletal organization, as given in Figure 8b. The DAPI‐stained nuclei across the coated sample were uniformly distributed and maintained a healthy elliptical morphology, with no signs of nuclear condensation or fragmentation indicative of early apoptosis. This uniformity of nuclear staining reflects stable adhesion and sustained metabolic activity during the culture period. The merged actin nucleus images further revealed that MSCs on the coated surface developed extensive F‐actin networks characterized by aligned, continuous stress fibers traversing the cytoplasm, a hallmark of physiologically healthy and well‐spread cells. The magnified single‐cell image reinforces this interpretation, showing a highly organized actin architecture with a centrally positioned, intact nucleus, consistent with healthy spreading and cytoskeletal organization. Compared to the control, the coated surfaces did not induce any adverse morphological changes; instead, MSCs displayed comparable or even more pronounced spreading behavior. The absence of rounded or retracted cells indicates that the coating did not trigger cytotoxic responses or inhibit integrin‐mediated adhesion, consistent with the viability data showing >90% metabolic activity across all groups as discussed in the previous section. These findings confirm the biocompatibility of the coated surfaces and highlight their potential to facilitate strong bone implant interactions in subsequent in vitro and in vivo applications.

### 4.4 Biofilm Formation on Implants

Biofilm formation on implant surfaces initiates with bacterial adhesion, followed by maturation into structured communities that are difficult to eradicate. In this study, biofilm burden on Ti64 surfaces was quantified by CFU enumeration after standardized washing and sonication of adherent bacteria, enabling direct comparison of bacterial attachment and biofilm formation across VED conditions and surface treatments [55]. Host cells such as fibroblasts and immune cells also attach to implants, and their distribution can either hinder or facilitate bacterial colonization [56]. Importantly, implant surface characteristics, including roughness, hydrophobicity, and charge, affect both bacterial adhesion and host cell spread; rougher surfaces tend to promote bacterial clustering, while smoother surfaces encourage more uniform host cell coverage, thereby limiting niches available for biofilm growth [57]. Once biofilms form, they produce extracellular polymeric substances that shield bacteria from immune attack, leading to an irregular distribution of immune cells around the implant and allowing biofilms to persist [58]. The competition between host cells and bacteria is critical: early host cell colonization can block bacterial adhesion and reduce biofilm formation, whereas bacterial dominance in cell distribution facilitates biofilm establishment and resistance to clearance [59]. Figure 9a represents representative agar plate images illustrating bacterial colony formation on Ti64 samples fabricated at different volumetric energy densities (VED: E107, E63, and E320) and subjected to polishing or etching followed by HAp/rGO coating, while Figure 9b provides the corresponding quantitative biofilm formation expressed as CFU ×10^5^ mL^−^^1^. A clear correlation is observed between the visual colony density in the plate images and the quantified CFU values, validating the consistency of the biofilm assessment.

**FIGURE 9:**
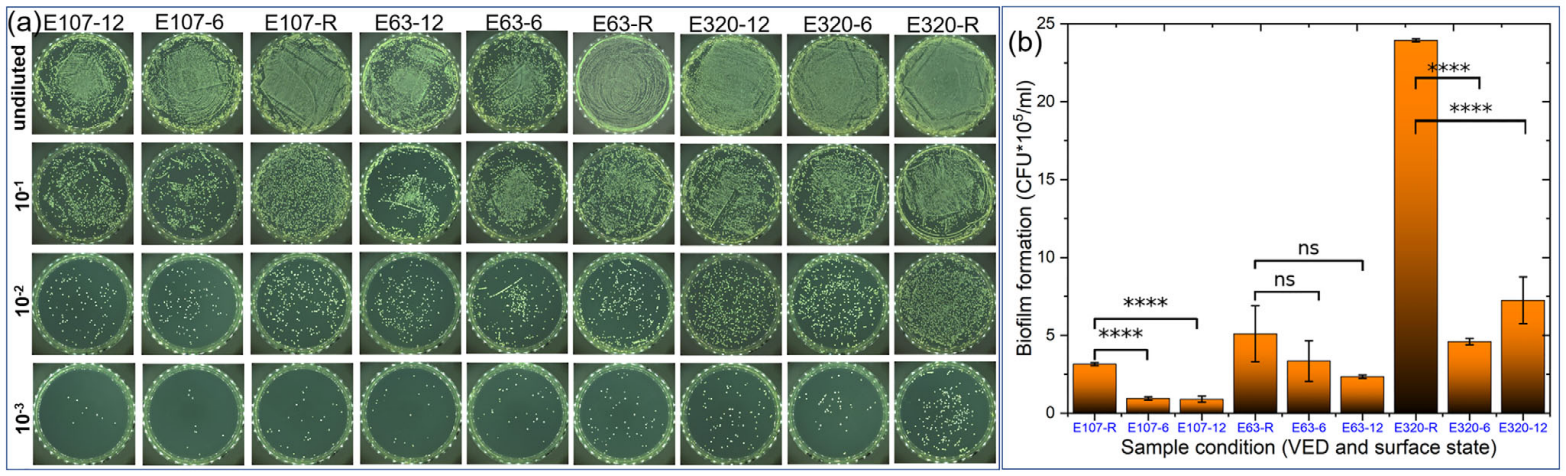
(a) Representative agar plate images showing bacterial biofilm formation on Ti64 samples fabricated at different volumetric energy densities (VED: E107, E63, and E320) and subjected to polishing (R) or acid etching (6 h and 12 h), followed by HAp/rGO coating. (b) Quantitative biofilm formation expressed as colony‐forming units (CFU ×10^5^ mL^−^^1^) for the corresponding sample conditions. All etched samples were treated under identical conditions and coated with HAp/rGO; error bars represent standard deviation. Data are mean ± SD (n = 3). Significance vs Reference was assessed using Welch's t‐test with Holm correction (* p < 0.05, ** p < 0.01, *** p < 0.001, **** p < 0.0001; ns, not significant).

For the polished and coated R samples, a strong dependence on VED is evident. The E320‐R condition exhibits dense and continuous bacterial colonies in Figure 9a, corresponding to the highest CFU value in Figure 9b, whereas the E107‐R sample shows comparatively fewer colonies and lower CFU counts. This indicates that, despite identical HAp/rGO coatings, the underlying substrate condition, as governed by VED, significantly influences bacterial adhesion and biofilm development. Such behavior is consistent with the known influence of SLM energy density on surface oxide thickness and chemical heterogeneity, which can adversely affect coating uniformity and limit the effective exposure of rGO at the surface–environment interface. Etched and coated samples (6 and 12 h) showed lower CFU values than the corresponding R controls. The reduction was significant for E107 (p < 0.0001) and E320 (p < 0.001), while E63 showed a non‐significant decreasing trend (p = 0.067) as given in Figure 9b. The plate images show more isolated and sparsely distributed colonies, indicating inhibited biofilm maturation. However, differences among VED conditions persist even after identical etching and coating treatments. Specifically, E107‐6/12 and E63‐6/12 samples consistently exhibit lower colony densities and CFU values than their E320 counterparts, demonstrating that VED‐dependent substrate characteristics continue to modulate antibacterial performance. Notably, extending the etching duration from 6 to 12 h does not eliminate the VED‐dependent trend, as E320‐12 still shows higher biofilm formation relative to lower‐VED samples. This suggests that the antibacterial effectiveness of the HAp/rGO coating is not solely determined by surface treatment but is strongly influenced by VED‐controlled factors, including near‐surface microstructure, oxide chemistry, and coating–substrate interfacial quality. The persistence of higher CFU values for E320 samples implies reduced functional exposure or effectiveness of rGO on high‐VED substrates. These results demonstrate that while HAp/rGO coating reduces biofilm formation across all treated samples, optimal antibacterial performance is achieved on substrates fabricated at lower and intermediate VEDs, emphasizing the importance of coupling additive manufacturing parameter selection with surface functionalization strategies. The VED‐dependent antibacterial response indicates that bacterial adhesion was influenced not only by the HAp/rGO coating composition but also by the substrate morphology generated during SLM processing. The coated E320 sample showed the highest biofilm formation despite the presence of rGO. This behavior may be associated with the excessive energy input used for E320, which can generate localized overheating, keyhole‐type surface features, deeper valleys, and defect‐associated regions. During coating deposition, these irregularities may promote nonuniform HAp/rGO accumulation and create localized microvoids or sheltered regions within the coating. Such features can protect bacteria from direct contact with the antibacterial rGO‐containing surface and provide favorable sites for bacterial attachment and biofilm retention. In contrast, the low‐ and intermediate‐VED samples showed significantly reduced bacterial adhesion after coating, suggesting that their surface morphology allowed more effective exposure and distribution of the HAp/rGO coating. Therefore, the antibacterial behavior of the coated samples resulted from the combined effects of rGO‐related antibacterial activity, VED‐dependent surface morphology, coating uniformity, and the presence of defect‐associated bacterial sheltering sites.

### 4.5 Discussion VED vs Bio‐compatibility

This study systematically investigated the combined influence of SLM processing parameters and biofunctional surface modification on the biological performance of Ti64 implants. The results demonstrate that VED plays a critical role in governing substrate microstructure, surface characteristics, and, consequently, the effectiveness of subsequent surface treatments. Despite identical acid etching and HAp/rGO coating conditions, biofilm formation and cell adhesion exhibited a strong dependence on VED, confirming that underlying SLM‐induced features significantly modulate biological response.

Lower and intermediate VED conditions promoted improved cell–surface interactions after HAp/rGO coating, with the E63 samples showing the most favorable cell attachment and surface coverage. In contrast, high‐VED samples exhibited inferior antibacterial performance, as evidenced by increased biofilm formation, even in the presence of the HAp/rGO coating. These findings indicate that excessive energy input during SLM can adversely affect coating functionality through substrate‐dependent surface chemistry and interfacial effects. Micro‐CT analysis further revealed increased porosity at low VED, while an optimum balance between densification and structural integrity was achieved at a VED of 107 J·mm^−^^3^.

The VED‐dependent biological and antibacterial responses should be interpreted by distinguishing between cell‐favorable hierarchical roughness and bacteria‐favorable sheltered defects. The improved cell–surface interaction observed for the lower‐VED E63 sample is likely related to its increased micro‐/nano‐scale surface features after HAp/rGO coating. These features can increase the effective surface area, improve coating retention, and provide more anchoring points for protein adsorption, focal adhesion formation, and MSC spreading. Since mammalian cells are much larger than bacteria and actively remodel their attachment sites through cytoskeletal organization, moderate surface irregularity and exposed bioactive HAp/rGO features can promote cell attachment. However, bacterial adhesion and biofilm formation are governed by different mechanisms. Bacteria are much smaller than MSCs and can colonize protected microvoids, deep valleys, interparticle gaps, and nonuniform coating regions. Therefore, the higher biofilm formation observed for the high‐VED E320 sample after coating is likely not due simply to higher roughness, but to the presence of high‐VED‐induced irregularities such as keyhole‐related depressions, remelted zones, deeper valleys, and localized defect‐associated regions. During HAp/rGO deposition, these regions may promote nonuniform coating accumulation and create sheltered sites where bacteria can be protected from direct contact with the antibacterial rGO‐containing surface. Such microenvironments can favor bacterial retention and biofilm development. Thus, the lower‐VED E63 surface can support improved MSC attachment because it provides accessible hierarchical roughness and better exposure of bioactive HAp/rGO features, while the high‐VED E320 surface can support higher biofilm formation because excessive‐energy‐induced defects may create protected bacterial niches and less uniform coating exposure.

Importantly, all coated samples exhibited cell viability exceeding 90%, satisfying ISO 10993‐5 non‐cytotoxicity criteria. The absence of cytotoxic effects was further confirmed by CCK‐8 assays and Phalloidin–DAPI staining, indicating that the applied surface treatments are safe and biocompatible, with no evidence of harmful ion or particle release. Overall, the combined use of optimized SLM parameters and HAp/rGO surface coating effectively enhances implant biofunctionality by promoting cell adhesion while suppressing bacterial biofilm formation.

From a broader perspective, the biological performance achieved in this study compares favorably with that typically reported for non‐coated commercial Ti64 implants, which predominantly rely on machined, polished, or grit‐blasted and acid‐etched surfaces to promote osseointegration. While such conventional surfaces are generally cytocompatible, they offer limited control over antibacterial functionality and remain susceptible to biofilm formation, particularly under clinically relevant conditions. In contrast, the present results demonstrate that integrating optimized SLM processing parameters with HAp/rGO surface functionalization enables simultaneous enhancement of cell adhesion and suppression of bacterial biofilm formation, two performance metrics that are often difficult to achieve concurrently in existing implant products. These results indicate that the proposed SLM–HAp/rGO strategy offers a multifunctional advantage over existing non‐coated implant surfaces by enabling controlled porosity, enhanced cell–material interactions, and effective biofilm mitigation within a single manufacturing framework.

Future studies should extend the present findings toward translational relevance by directly benchmarking non‐coated and commercially available Ti64 implant surfaces under identical biological and microbiological testing conditions. Long‐term in vivo investigations are required to evaluate coating durability, ion release stability, and sustained antibacterial performance under physiologically relevant loading and fluid‐flow environments. In addition, systematic optimization of HAp/rGO composition, coating thickness, and graphene distribution should be pursued to further enhance the balance between osteogenic response and biofilm inhibition. From a manufacturing perspective, expanding the VED window and correlating process‐induced microstructural features with coating–substrate interfacial behavior would enable more precise control of biofunctional outcomes. Finally, integration of computational modeling or data‐driven approaches to predict the combined effects of SLM parameters and surface functionalization on biological performance could accelerate the design of next‐generation, infection‐resistant orthopedic implants.

## 5 Conclusion

This study systematically clarifies how SLM processing and biofunctional surface engineering must be co‐designed to achieve reliable biological performance in Ti64 implants. VED was shown to be a decisive upstream variable that shapes substrate microstructure, porosity, and surface/interfacial characteristics. Although all coated conditions remained highly cytocompatible, exhibiting >90% cell viability, cell adhesion and bacterial biofilm outcomes depended strongly on VED, confirming that SLM‐induced features substantially modulate cell–material and bacteria–surface interactions. Lower to intermediate VED promoted more favorable post‐coating cell attachment and surface coverage, with E63 providing the most pronounced improvement in cell–surface interactions, whereas excessive energy input (high VED) diminished antibacterial efficacy, evidenced by elevated biofilm formation despite the presence of HAp/rGO, likely due to VED‐driven changes in surface chemistry and coating–substrate interfacial behavior. Compared with conventional non‐coated commercial Ti64 surfaces, which mainly support osseointegration but offer limited antibacterial control, the integrated SLM–HAp/rGO strategy provides a multifunctional pathway to simultaneously promote cellular integration and suppress biofilm formation within a single manufacturing framework.

## Author Contributions

**Muhammad Usama Zaheer**: Conceptualization, Visualization, Data Curation, Image design, Methodology, Writing – review & editing, Writing – original draft and revision. **Vahid Jahed**: Writing – review & editing, Visualization, Investigations and Methodology. **Rajveer Singh Rajaura**: XPS measurements and Writing – review & editing. **Fatemeh Rastegar Adib**: Data Curation, Image design and Methodology. **Muhammad Hassan Razzaq**: Visualization and Review. **Gokhan Demirel**: Investigation and Review. **Mobarak Abu Mraheil**: Data Curation, Image design and Methodology. **Thaqif El Khassawna**: Data Curation, Writing – review & editing, Writing – original draft and revision and Resources. **Mehmet Fatih Aycan**: Conceptualization, Visualization, Supervision, Writing – review & editing, Writing – original draft and revision, Funding acquisition and Resources. **Yogendra Kumar Mishra**: Conceptualization, Visualization, supervision, Writing – review & editing, Writing – original draft and revision, Funding acquisition and Resources.

## Ethics Approval and Consent to Participate

All procedures involving human participants were performed in accordance with the ethical standards of the Ethics Committee of the Faculty of Medicine, Justus Liebig University Giessen, and with the 1964 Declaration of Helsinki and its later amendments or comparable ethical standards. The study protocol was approved by the Ethics Committee of the Faculty of Medicine, Justus Liebig University Giessen (Approval No. 79/17, approved on June 20, 2017). Written informed consent was obtained from all participants prior to the collection of femoral head bone samples.

## Conflicts of Interest

The authors declare that they have no competing interests. The authors acknowledge the use of an AI‐assisted chatbot (e.g., ChatGPT, DeepSeek Chat) for limited purposes such as grammar refinement, language polishing, and initial structuring of non‐technical text. The AI tool was not employed for data analysis, scientific interpretation, or generating original technical content.

## Supporting information




**Supporting File**: smll75211‐sup‐0001‐SuppMat.docx.

## Data Availability

The data that support the findings of this study are available on request from the corresponding author. The data are not publicly available due to privacy or ethical restrictions.
