# Supplementary material for: Hierarchically Rough rGO/HAp Nanocoatings on SLM Ti–6Al–4 V Implants for Enhanced Biointegration
Source: Small. 2026 Aug 21;22(58):e75211. doi: 10.1002/smll.75211 (PMC13676828; doi:10.1002/smll.75211)
Supplement: Supplementary file 1 [file SMLL-22-e75211-s001.docx]

Supplementary Information

Hierarchically Rough rGO/HAp Nanocoatings on SLM Ti–6Al–4V Implants for Enhanced Biointegration

***Muhammad Usama Zaheer*, Vahid Jahed, Rajveer Singh Rajaura, Fatemeh Rastegar Adib, Muhammad Hassan Razzaq, Gökhan Demirel, Mobarak Abu Mraheil, Thaqif El Khassawna*, Mehmet Fatih Aycan*, Yogendra Kumar Mishra****

Dr. M. U. Zaheer, Dr. M. H. Razzaq, Prof. Y. K. Mishra

Smart Materials, NanoSYD, Mads Clausen Institute

University of Southern Denmark

Alsion 2, DK-6400, Sønderborg, Denmark

Email: [zaheer@mci.sdu.dk](mailto:musama.zaheer@gazi.edu.tr), [mishra@mci.sdu.dk](mailto:mishra@mci.sdu.dk)

Dr. M. U. Zaheer, Dr. M. H. Razzaq, Dr. M. F. Aycan

Department of Mechanical Engineering

Institute for Graduate School of Natural and Applied Sciences,

Gazi University, Ankara 06570, Türkiye

Email: [musama.zaheer@gazi.edu.tr](mailto:musama.zaheer@gazi.edu.tr), [mfaycan@gazi.edu.tr](mailto:mfaycan@gazi.edu.tr)

Dr. M. U. Zaheer, Dr. M. H. Razzaq, Dr. M. F. Aycan

Additive Manufacturing Technologies Application and Research Center - EKTAM,

Gazi University, Ankara 06570, Türkiye

Email: [musama.zaheer@gazi.edu.tr](mailto:musama.zaheer@gazi.edu.tr), [mfaycan@gazi.edu.tr](mailto:mfaycan@gazi.edu.tr)

R. S. Rajaura

School of Engineering and Materials Science

Queen Mary University of London

Mile End Rd, London E1 4NS, UK

F R Adib, Dr. V. Jahed, Prof. T. E. Khassawna

Experimental Trauma Surgery, Faculty of Medicine

Justus-Liebig-University, Giessen, Germany

Email:[thaqif.elkhassawna@chiru.med.uni-giessen.de](mailto:thaqif.elkhassawna@chiru.med.uni-giessen.de)

Dr. G. Demirel

Department of Chemistry, Faculty of Science, Gazi University

Ankara, 06500, Türkiye

FR Adib, Dr. MA Mraheil

Institute for Medical Microbiology, Justus-Liebig-University

Frankfurter Strasse 107, 35392 Giessen, Germany

**Keywords:** Orthopedic SLM implants; rGO/HAp nanocoating, Hierarchical morphology; Biointegration; Cytocompatibility

**Supplementary Figure 1 (S1)**

**Figure S1:** SLM-fabricated sample plate containing parameter-set specimens. Four distinct processing parameter sets were used to fabricate the samples (28 samples for each parameter set), which were subsequently employed in the experimental investigations described in this work.

**Supplementary Figure 2 (S2)**

**Figure S2**: Schematic representation of the overall experimental process: (a) selective laser melting (SLM) setup used for the fabrication of Ti-6Al-4V substrates under an inert gas atmosphere, (b) electrophoretic deposition (EPD) configuration employed for HAp/rGO composite coating formation under an applied electric field, and (c) post-deposition heat treatment in an argon environment to enhance coating adhesion and phase stability.

**Supplementary Figure 3 (S3)**

**Figure S3**: FESEM surface morphology of sample after 12-hour etching.

**Supplementary Figure 4 (S4)**

**Figure S4:** FESEM surface morphology of sample after 18-hour etching.

**Supplementary Figure 5 (S5)**

**Figure S5**: FESEM surface morphology of sample after 24-hour of etching.

**Supplementary Figure 6 (S6)**

**Figure S6:** Three-dimensional micro-CT reconstruction and defect mapping of sample E107.

**Supplementary Figure 7 (S7)**

**Figure S7:** Three-dimensional micro-CT reconstruction and defect mapping of sample E200.

**Supplementary Figure 8 (S8)**

**Figure S8:** Three-dimensional micro-CT reconstruction and defect mapping of sample E320.

**Supplementary Figure 9 (S9)**

**Figure S9:** Three-dimensional micro-CT reconstruction and defect mapping of sample E63.

**Supplementary Figure 10 (S10)**

**Figure S10: Equivalent diameter and cumulative volume fraction graphs indication D80 values of all concerned samples**: (e) Representative graph for sample E107 (f) Representative graph for sample E200 (g) Representative graph for sample E320 (h) Representative graph for sample E63.

**Supplementary Figure 11 (S11)**

**Figure S11:** Representative progressive-load scratch-test analysis of HAp/rGO-coated Ti64 samples. Full scratch-track overview, magnified images showing L_c1_ cohesive cracking, L_c2_ initial delamination, and L_c3_ complete coating removal, together with the corresponding friction-force and normal-load curves as a function **of scratch length.**

**Supplementary Figure 12 (S12)**

**Figure S12**: EDS spectra of Ti-6Al-4V samples.

**Supplementary Figure 13 (S-13).**

**Figure S13:** XPS spectra of Ti-6Al-4V samples.

**Supplementary Table TS-1**

Cell viability (%) of MSCs cultured on SLM-fabricated Ti64 samples from different VED groups (E107, E200, E320, E63) under pristine, etched, and etched + rGO/HAp-coated conditions after 24 h and 48 h exposure. Values represent mean ± standard deviation (SD). Control represents cells cultured without material contact.

| **S No** | **Exposure  time (Hrs)** | **Viability (%)** | **SD** | **VED group** | **Treatment** |
| --- | --- | --- | --- | --- | --- |
| 1 | 24 | 99 | 1.58 | Control | Control |
| 2 | 24 | 94.8 | 3.63 | E107 | Pristine |
| 3 | 24 | 95.16 | 2.88 | E63 | Pristine |
| 4 | 24 | 95 | 3.48 | E200 | Pristine |
| 5 | 24 | 93.94 | 3.46 | E320 | Pristine |
| 6 | 24 | 94.14 | 2.86 | E107 | Etched-6h + Coated |
| 7 | 24 | 94.12 | 3.15 | E63 | Etched-6h + Coated |
| 8 | 24 | 94.78 | 3.65 | E200 | Etched-6h + Coated |
| 9 | 24 | 95.36 | 2.81 | E320 | Etched-6h + Coated |
| 10 | 24 | 94.04 | 2.99 | E107 | Etched-12h + Coated |
| 11 | 24 | 93.82 | 2.94 | E63 | Etched-12h + Coated |
| 12 | 24 | 94.38 | 2.96 | E200 | Etched-12h + Coated |
| 13 | 24 | 95.12 | 2.71 | E320 | Etched-12h + Coated |
| 14 | 48 | 100.8 | 3.03 | Control | Control |
| 15 | 48 | 97.08 | 3.61 | E107 | Pristine |
| 16 | 48 | 98.08 | 3.71 | E63 | Pristine |
| 17 | 48 | 97.32 | 3.28 | E200 | Pristine |
| 18 | 48 | 96.72 | 3.3 | E320 | Pristine |
| 19 | 48 | 97.06 | 4.26 | E107 | Etched-6h + Coated |
| 20 | 48 | 96.38 | 2.9 | E63 | Etched-6h + Coated |
| 21 | 48 | 98.64 | 3.21 | E200 | Etched-6h + Coated |
| 22 | 48 | 96.4 | 2.8 | E320 | Etched-6h + Coated |
| 23 | 48 | 97.54 | 2.61 | E107 | Etched-12h + Coated |
| 24 | 48 | 97.66 | 3.66 | E63 | Etched-12h + Coated |
| 25 | 48 | 98.3 | 4.25 | E200 | Etched-12h + Coated |
| 26 | 48 | 97.66 | 3.05 | E320 | Etched-12h + Coated |

**Supplementary Table TS-2**

Summary of FTIR peak positions (cm⁻¹) and corresponding vibrational assignments/functional groups identified from the spectrum.

| **Peak position (cm⁻¹)** | **Assignment (vibration mode)** | **Functional group / species** |
| --- | --- | --- |
| 850 | Ti–O vibrations | Ti–O / Ti–O–Ti (titanium oxide lattice) |
| 1050 | C–O stretch | C–O (often surface carbonate/organic residue-related) |
| 1630 | H–O–H bending | Adsorbed water (H₂O) |
| 2350 | CO₂ asymmetric stretch | CO₂ (typically atmospheric/adsorbed CO₂) |
| 3500 | O–H stretch | Surface hydroxyls / adsorbed water (–OH) |

**Supplementary Table TS-3**

Elemental peak assignments from the XPS survey spectrum (binding energy, eV) for the analyzed surface, indicating the presence of O, Ti, C, Ca, P, and Al.

| **Peak (label)** | **Typical binding energy (eV)** | **Assignment** |
| --- | --- | --- |
| O 1s | ~530–532 | Oxygen (oxide / hydroxyl / adsorbed oxygen species) |
| Ti 2p | ~458–465 | Titanium (Ti–O / TiO₂-related states) |
| C 1s | ~284.8–286 | Adventitious carbon / surface carbon species |
| Ca 2p | ~347–352 | Calcium (e.g., Ca–O / Ca–phosphate environments) |
| P 2p | ~132–135 | Phosphorus (phosphate, PO₄³⁻) |
| Al 2p | ~73–75 | Aluminum (Al–O / alumina-related states) |
